# Nanoscale thermodynamics needs the concept of a disjoining chemical potential

**DOI:** 10.1038/s41467-023-36970-7

**Published:** 2023-04-01

**Authors:** W. Dong

**Affiliations:** 1 Laboratoire de Chimie, CNRS, UMR 5182, Ecole Normale Supérieure de Lyon, 46, Allée d’Italie, 69364 Lyon, Cedex 07 France; 2 State Key Laboratory of Chem/Biosensing and Chemometrics, College of Chemistry and Chemical Engineering, Hunan University, 410082 Changsha, China

**Keywords:** Thermodynamics, Thermodynamics

## Abstract

Disjoining pressure was discovered by Derjaguin in 1930’s, which describes the difference between the pressure of a strongly confined fluid and the corresponding one in a bulk phase. It has been revealed recently that the disjoining pressure is at the origin of distinct differential and integral surface tensions for strongly confined fluids. Here we show how the twin concept, disjoining chemical potential, arises in a reminiscent way although it comes out eighty years later. This twin concept advances our understanding of nanoscale thermodynamics. Ensemble-dependence (or environment-dependence) is one hallmark of thermodynamics of small systems. We show that integral surface tension is ensemble-dependent while differential surface tension is not. Moreover, two generalized Gibbs-Duhem equations involving integral surface tensions are derived, as well as two additional adsorption equations relating surface tensions to adsorption-induced strains. All the results obtained in this work further evidence that an approach alternative of Hill’s nanothermodynamics is possible, by extending Gibbs surface thermodynamics instead of resorting to Hill’s replica trick. Moreover, we find a compression-expansion hysteresis without any underlying phase transition.

## Abstract (web summary)

Matter behaves differently at the nanoscale. Here, the author introduces the concept of a disjoining chemical potential for nanoscale thermodynamics, showing that thermodynamic functions depend on the environment, and suggests possible experimental verifications.

## Introduction

Thermodynamics provides a robust framework applicable not only for fundamental sciences but also for technological innovations. It is quite like a jigsaw puzzle, a few missing pieces prevent figuring out the whole picture. For thermodynamics of macroscopic systems, the main pieces are now in place, giving essentially a whole picture. Terrell L. Hill proposed a first thermodynamic approach for dealing with small systems^1,2^, named now as nanothermodynamics^3^, which is attracting much renewed attention with the advent of nanoscience and nanotechnology. Its foundation and various fundamental aspects are currently under active scrutiny^4–18^. Hill’s nanothermodynamics is based on a replica trick and a new thermodynamic function, subdivision potential, which is conjugated with replica number. However, it has never been measured experimentally, this constitutes a major obstacle for the experimental validation of Hill’s theory. Moreover, the abstract character of the subdivision potential prevents perceiving directly some consequences due to size smallness. For example, Hill insisted rightly on the environment-dependence (or ensemble-dependence) and the necessity to distinguish two types of intensive thermodynamic variables, i.e., differential and integral ones, for describing small systems. However, it remains elusive to relate the ensemble-dependent subdivision potentials to any experimental observables. The environment-dependence may have various implications. Does it mean that some thermodynamic potentials are no longer applicable for small systems, or, they can yield different values for some deduced quantities? For example, is the chemical potential calculated from Helmholtz free energy identical to that from Gibbs free energy? For thermodynamics of macroscopic systems, different thermodynamic potentials can be transformed from each other with Legendre transforms. Do these transforms still hold? Even if they do, which type of intensive variables should be used for the Legendre transforms, differential or integral ones? In this work, all these issues will be investigated in details.

One salient feature of a small system is its dominant or even overwhelming surface contribution to thermodynamic potentials, thus accounting adequately for it plays a primordial role. We showed recently that when surface region invades the whole space in a nanoscale system, Gibbs surface thermodynamics has to be extended to account for distinct differential and integral surface tensions^19^. The origin of different differential and integral surface tensions, found with a thermodynamic formulation based on grand potential^19^, can be traced to Derjaguin’s disjoining pressure. In this work, we reveal another concealed face of the integral surface tension, intimately connected to disjoining chemical potential (a twin concept of disjoining pressure). This finding adds one missing piece to the jigsaw puzzle of nanoscale thermodynamics and advances our understanding of the whole picture.

Despite its discovery in 1930’s by Derjaguin and its experimental measurements since 1950’s (see, e.g. refs. ^20–22^ and the references therein), disjoining pressure does not seem to have entered into the basic knowledge of thermodynamics at an equal footing of the other thermodynamic functions, such as pressure, chemical potential or surface tension. From statistical-mechanics, it is established that an intensive thermodynamic variable of a system embedded in a reservoir, e.g., pressure, has the same value as that of the reservoir, if the conjugated extensive variable, e.g., volume, is allowed to fluctuate. In a grand canonical ensemble, the chemical potential of the considered system is equal to that of the reservoir since the conjugated variable, particle number, is allowed to fluctuate. If the considered system is a macroscopic one, its pressure is also equal to that of the reservoir although its volume is fixed in the grand canonical ensemble. Derjaguin evidenced first that if the size of a system is reduced sufficiently in one direction, the pressure of the system in this direction ceases to be equal to that of the reservoir. He also coined the term disjoining pressure for the pressure difference between the system and the reservoir. Only very recently, is it revealed that the existence of two distinct surface tensions, i.e., differential and integral ones, is intimately related to the disjoining prerssure^19^. Derjaguin’s disjoining pressure is the only known disjoining thermodynamic function up to now. The disjoining chemical potential to be revealed in the present work is the second one. Disjoining thermodynamics functions arise when it is impossible to delineate clearly a surface region from the rest of a system. They account for the intertwined volume and surface contributions to thermodynamic potentials. The prototypes for such a situation can be thin slice-shape systems or fluids confined in a narrow slit pore. Here, we limit our investigation to systems bounded between two flat surfaces (i.e., no contribution of surface curvatures). Nevertheless, disjoining chemical potential can arise also in more general situations, e.g. a fluid confined between a solid sphere closely approached to a flat solid surface (experimental situation for measurements with an atomic force microscope).

Our current knowledge about hysteresis is largely based on the experimental observations and theoretical investigations of phase transitions. One can hardly imagine any hysteresis without an underlying phase transition. This article proposes a compression-expansion hysteresis without any underlying phase transition.

## Results

### Disjoining chemical potential: the twin of disjoining pressure

Disjoining pressure can arise in the thermodynamic formulation based on grand potential with *T*, *V*, $\mu$, ${{{{{\mathcal{A}}}}}}$ as independent variables (*T*: temperature; *V*: volume; $\mu$: chemical potential; ${{{{{\mathcal{A}}}}}}$: total surface area). In this case, the temperature and chemical potential of the system are respectively the same as those of the reservoir thanks to the exchange of thermal energy and particles between the system and the reservoir. However, the pressures of the system and the reservoir can be different under some conditions since the volume is not allowed to fluctuate. Disjoining pressure arises when the size of the system is sufficiently reduced in one direction, e.g., a system of thin slice shape. If we replace $\mu$ and *V* by their respective conjugated variables, i.e., $N$ and *p*, no disjoining pressure is possible due to the volume adjustment to keep the same pressure in the considered small system and its reservoir. But, what happens in such a *pTN*-ensemble under the similar circumstances when a disjoining pressure arises in a grand canonical ensemble? One can plausibly imagine that a disjoining chemical potential can arise in the absence of particle exchange with the reservoir. We show below this indeed happens. For a *pTN*-ensemble, Gibbs free energy is the thermodynamic potential and described by the following fundamental equation,

1 $${dG}=-{SdT}+{Vdp}+\mu {dN}+\gamma d{{{{{\mathcal{A}}}}}},$$

where the differential chemical potential, *µ*, differential surface tension, *γ*, volume, *V*, and entropy, *S*, are defined respectively by

2 $$\mu={\left(\frac{\partial G}{\partial N}\right)}_{T,p{{{{{\mathscr{,}}}}}}{{{{{\mathcal{A}}}}}}},$$

3 $$\gamma={\left(\frac{\partial G}{\partial {{{{{\mathcal{A}}}}}}}\right)}_{T,p,N},$$

4 $$V={\left(\frac{\partial G}{\partial p}\right)}_{T,N{{{{{\mathscr{,}}}}}}{{{{{\mathcal{A}}}}}}},$$

5 $$S=-{\left(\frac{\partial G}{\partial T}\right)}_{p,N{{{{{\mathscr{,}}}}}}{{{{{\mathcal{A}}}}}}}.$$

At this point, it is useful to recall the definition of differential and integral intensive thermodynamic functions (terminology coined by Hill^1,2^). A differential intensive function is given by the derivative of a thermodynamic potential with respect to an extensive variable. For example, the derivative of *G* with respect to *N* gives the differential chemical potential. For macroscopic systems, it is well-known that the quotient, $G{N}^{-1}$, gives also chemical potential and it is equal to that given by the derivative. For small systems, it is necessary to distinguish such two types of intensive quantities since they are no longer equal. Following Hill, we keep the same symbols for the differential thermodynamic quantities as those used for the thermodynamics of macroscopic systems while a hat is added for the integral intensive thermodynamic quantities, defined as the quotient between two extensive variables, e.g., $\hat{\mu }=G{N}^{-1}$ while the differential chemical potential is defined by Eq. (2). Although Hill’s denotation is used, we do not resort to his replica trick, neither to his nanothermodynamics. Despite some similarity between our and Hill’s definitions of differential intensive variables, they are not identical since neither subdivision potential nor replica number appears in our formulation based on the usual surface thermodynamics as differential intensive variables are concerned. When the surface contribution becomes dominant or overwhelming, every extensive thermodynamic function scales with surface area. Our previous study^19^ showed that for a fixed slit-pore width, the volume and the grand potential scale indeed with surface area. For the *pTN*-ensemble considered here, we have,

6 $$G\left(T,\,p,\,\lambda N,\,\lambda {{{{{\mathcal{A}}}}}}\right)=\lambda G\left(T,\,p,\,N{{{{{\mathscr{,}}}}}}\, {{{{{\mathcal{A}}}}}}\right),$$

thus

7 $$G\left(T,\, p,\, N{{{{{\mathscr{,}}}}}}\, {{{{{\mathcal{A}}}}}}\right)=\mu N+\gamma {{{{{\mathcal{A}}}}}},$$

and the following Gibbs-Duhem equation holds,

8 $${SdT}-{Vdp}+{Nd}\mu {{{{{\mathscr{+}}}}}}{{{{{\mathcal{A}}}}}}d\gamma=0.$$

From Eq. (7), we obtain immediately the following result for integral chemical potential,

9 $$\hat{\mu }=\frac{G\left(p,\,T,\, N{{{{{\mathscr{,}}}}}}\, {{{{{\mathcal{A}}}}}}\right)}{N}=\mu+\gamma \frac{{{{{{\mathcal{A}}}}}}}{N}.$$

We also obtain straightforwardly the following relation between differential and integral chemical potentials,

10 $$\mu=\hat{\mu }+N{\left(\frac{\partial \hat{\mu }}{\partial N}\right)}_{T,p{{{{{\mathscr{,}}}}}}{{{{{\mathcal{A}}}}}}}.$$

Equation (10) is reminiscent of the relation between differential and integral pressures or that between differential and integral surface tensions^19^. When *N* scales with ${{{{{\mathcal{A}}}}}}$ and the second term on the right-hand-side (RHS) of Eq. (9) is nonnegligible, we have $\hat{\mu }\, \ne \, \mu$. This is one characteristic distinguishing the thermodynamics for small or macroscopic systems. Without resorting to Hill’s replica trick, we show here how differential and integral chemical potentials become different.

If every thermodynamic potential is equally valid for describing small systems, we anticipate that a disjoining chemical potential (i.e., ${\varpi=\mu -\mu }^{{bulk}}\, \ne \, 0$) can arise in a *pTN*-ensemble reminiscently as the disjoining pressure does in a grand canonical ensemble. In this case, we rewrite Eq. (7) in terms of a bulk contribution and a surface one as follows,

11 $$G\left(T,\, p,\, N{{{{{\mathscr{,}}}}}}\, {{{{{\mathcal{A}}}}}}\right)={\mu }^{{bulk}}\left(T,\, p\right)N+\varpi N+\gamma {{{{{\mathcal{A}}}}}}{{{{{\mathscr{=}}}}}}{\mu }^{{bulk}}\left(T,\, p\right)N+{\hat{\gamma }}_{G}{{{{{\mathcal{A}}}}}},$$

where ${\mu }^{{bulk}}\left(T,\, p\right)$ is the chemical potential of the same system in the bulk and ${\hat{\gamma }}_{G}$, is an integral surface tension defined by,

12 $${\hat{\gamma }}_{G}=\gamma+\varpi \frac{N}{{{{{{\mathcal{A}}}}}}}.$$

The index *G* indicates this integral surface tension is defined from Gibbs free energy. It will be shown that although differential surface tension, $\gamma$, is ensemble-independent (thus without any index), the integral surface tension is ensemble-dependent. From Eq. (11), we derive also readily the following relation between $\gamma$ and ${\hat{\gamma }}_{G}$,

13 $$\gamma={\left(\frac{\partial G}{\partial {{{{{\mathcal{A}}}}}}}\right)}_{T,p,N}={\left[\frac{\partial \left({{{{{\mathcal{A}}}}}}{\hat{\gamma }}_{G}\right)}{\partial {{{{{\mathcal{A}}}}}}}\right]}_{T,p,N}={\hat{\gamma }}_{G}{{{{{\mathscr{+}}}}}}{{{{{\mathcal{A}}}}}}{\left[\frac{\partial {\hat{\gamma }}_{G}}{\partial {{{{{\mathcal{A}}}}}}}\right]}_{T,p,N},$$

which is similar to the analogue relation found previously in a grand canonical ensemble^19^, i.e.,

14 $$\gamma={\left(\frac{\partial \Omega }{\partial {{{{{\mathcal{A}}}}}}}\right)}_{T,\mu,V}={\left[\frac{\partial \left({{{{{\mathcal{A}}}}}}{\hat{\gamma }}_{\Omega }\right)}{\partial {{{{{\mathcal{A}}}}}}}\right]}_{T,\mu,V}={\hat{\gamma }}_{\Omega }{{{{{\mathscr{+}}}}}}{{{{{\mathcal{A}}}}}}{\left[\frac{\partial {\hat{\gamma }}_{\Omega }}{\partial {{{{{\mathcal{A}}}}}}}\right]}_{T,\mu,V},$$

where $\Omega$ is grand potential. ${\hat{\gamma }}_{\Omega }$ (index $\Omega$ distinguishing it from ${\hat{\gamma }}_{{{{{{\rm{G}}}}}}}$) is the integral surface tension defined by,

15 $$\Omega \left(T,\, \mu,\, V{{{{{\mathscr{,}}}}}}\, {{{{{\mathcal{A}}}}}}\right)={-p}^{{bulk}}\left(T,\, \mu \right)V+{\hat{\gamma }}_{\Omega }{{{{{\mathcal{A}}}}}}.$$

In Eq. (15), ${p}^{{bulk}}\left(T,\mu \right)$ is the pressure of the corresponding bulk system at the same *T* and *µ*. Equation (12) shows that ${\hat{\gamma }}_{G}$ depends on $N{{{{{{\mathcal{A}}}}}}}^{-1}$ (surface density). This reflects precisely the impossible delineation of a clear surface region in the whole system. We see here ${\hat{\gamma }}_{G}\, \ne \,\gamma$, when $\varpi \, \ne \, 0$, in a reminiscent way as what happens in a grand canonical ensemble, i.e., ${\hat{\gamma }}_{\Omega }\, \ne \, \gamma$, when $\Pi \, \ne \, 0$ ($\Pi$: disjoining pressure)^19^. So, disjoining chemical potential is a twin concept of disjoining pressure although it comes out now more than eighty years later. Taking account of the fact that *µ* and *γ* satisfy the Gibbs-Duhem equation given in Eq. (8) and taking the derivative on the both sides of Eq. (12) with respect to the surface density, $\hat{\varphi }=N{{{{{{\mathcal{A}}}}}}}^{-1}$, (the hat emphasizing it is an integral intensive variable), we obtain,

16 $${\left[\frac{\partial {\hat{\gamma }}_{G}}{\partial \hat{\varphi }}\right]}_{T,p}=\varpi .$$

Substituting Eq. (16) back into Eq. (12), we obtain,

17 $$\gamma={\hat{\gamma }}_{G}-\hat{\varphi }{\left[\frac{\partial {\hat{\gamma }}_{G}}{\partial \hat{\varphi }}\right]}_{T,p}.$$

Although this relation is compatible with Eq. (13), it provides a more practical description since ${\hat{\gamma }}_{G}$ is a function of *T*, *p* and $\hat{\varphi }$, but does not depend on *N* and ${{{{{\mathcal{A}}}}}}$ separately.

The well-known Gibbs-Duhem equation holds for differential intensive variables (see Eq. 8). Now, we show a generalized Gibbs-Duhem equation for the integral surface tension, ${\hat{\gamma }}_{G}$ can be derived also. Subtracting the bulk Gibbs-Duhem equation with the same *N* at the same *p* and *T* from Eq. (8) and using Eq. (12) to express $\gamma$ in term of ${\hat{\gamma }}_{{{{{{\rm{G}}}}}}}$ and $\varpi$, we obtain,

18 $$\left(S-{S}^{{bulk}}\right){dT}-\left(V-{V}^{{bulk}}\right){dp}{{{{{\mathscr{-}}}}}}{{{{{\mathcal{A}}}}}}\varpi d\hat{\varphi }{{{{{\mathscr{+}}}}}}{{{{{\mathcal{A}}}}}}d{\hat{\gamma }}_{G}=0,\,{{{{{\rm{for}}}}}}\; p={p}^{{bulk}},\,\varpi \, \ne \, 0.$$

In a similar way, we can derive the following Gibbs-Duhem equation for ${\hat{\gamma }}_{\Omega }$,

19 $$\left(S-{S}^{{bulk}}\right){dT}+\left(N-{N}^{{bulk}}\right)d\mu {{{{{\mathscr{+}}}}}}{{{{{\mathcal{A}}}}}}\Pi d\hat{{{{{{\mathscr{l}}}}}}}{{{{{\mathscr{+}}}}}}{{{{{\mathcal{A}}}}}}d{\hat{\gamma }}_{\Omega }=0,\, {{{{{\rm{for}}}}}}\, \mu={\mu }^{{bulk}},\,\Pi \, \ne \, 0,$$

where $\hat{{{{{{\mathscr{l}}}}}}}=V{{{{{{\mathcal{A}}}}}}}^{-1}$ and the following relation between $\gamma$ and ${\hat{\gamma }}_{\Omega }$ was used^19^,

20 $${\hat{\gamma }}_{\Omega }=\gamma -\Pi \frac{V}{{{{{{\mathcal{A}}}}}}}=\gamma -\Pi \hat{{{{{{\mathscr{l}}}}}}}.$$

From Eq. (18), we derive readily the following equation for ${\hat{\gamma }}_{G}$,

21 $${\left(\frac{\partial {\hat{\gamma }}_{G}}{\partial p}\right)}_{T,\hat{\varphi }}=\hat{\Delta },$$

where

22 $$\hat{\Delta }=\frac{V-{V}^{{bulk}}}{{{{{{\mathcal{A}}}}}}}.$$

We name $\hat{\Delta }$ as integral adsorption strain, which is the thickness difference between the inhomogeneous system and its bulk counterpart with same *N*, ${{{{{\mathcal{A}}}}}}$, *T* and *p*. Due to its close analogy to the generalized Gibbs adsorption equation derived in ref. ^19^, we call Eq. (21) also a generalized Gibbs adsorption equation although it relates ${\hat{\gamma }}_{G}$ to the adsorption strain, $\hat{\Delta }$.

If we define the differential adsorption strain, $\Delta$, by,

23 $$\Delta=\hat{\Delta }-\hat{\varphi }{\left[\frac{\partial \hat{\Delta }}{\partial \hat{\varphi }}\right]}_{T,p},$$

which is an analogue of Eq. (17). From Eq. (22), we have,

24 $$\hat{\Delta }=\frac{V-{V}^{{bulk}}}{{{{{{\mathcal{A}}}}}}}	=\frac{1}{{{{{{\mathcal{A}}}}}}}\left[{\left(\frac{\partial G}{\partial p}\right)}_{T,\,N{{{{{\mathscr{,}}}}}}\,{{{{{\mathcal{A}}}}}}}-{\left(\frac{\partial {G}^{{bulk}}}{\partial p}\right)}_{T,\,N}\right] \\ 	=\hat{\varphi }{\left(\frac{\partial \mu }{\partial p}\right)}_{T,\hat{\varphi }}+{\left(\frac{\partial \gamma }{\partial p}\right)}_{T,\hat{\varphi }}-{\hat{\varphi }\left(\frac{\partial {\mu }^{{bulk}}}{\partial p}\right)}_{T} \\ 	={\hat{\varphi }\left(\frac{\partial \varpi }{\partial p}\right)}_{T,\hat{\varphi }}+{\left(\frac{\partial \gamma }{\partial p}\right)}_{T,\hat{\varphi }}$$

Equation (7) is used when going to the third equality in Eq. (24). Substituting Eq. (24) into Eq. (23) and noting that $\gamma$ and $\mu$ satisfy the Gibbs-Duhem equation given in Eq. (8) and *μ*^*bulk*^ does not depend on $\hat{\varphi }$, we obtain the following generalized adsorption equation for the differential surface tension,

25 $${\left(\frac{\partial \gamma }{\partial p}\right)}_{T,\hat{\varphi }}=\Delta .$$

All the above results rely on the assumption that Gibbs free energy describes small systems as well as grand potential. Caution must be taken here because of a dualism behind this assumption. It seems to affirm ensemble-equivalence, but we will show it holds only for differential intensive variables (i.e., same in all the ensembles). However, integral intensive variables are ensemble-dependent, i.e., different in different ensembles.

### Illustration with exact statistical-mechanics results

**Fig. 1:**
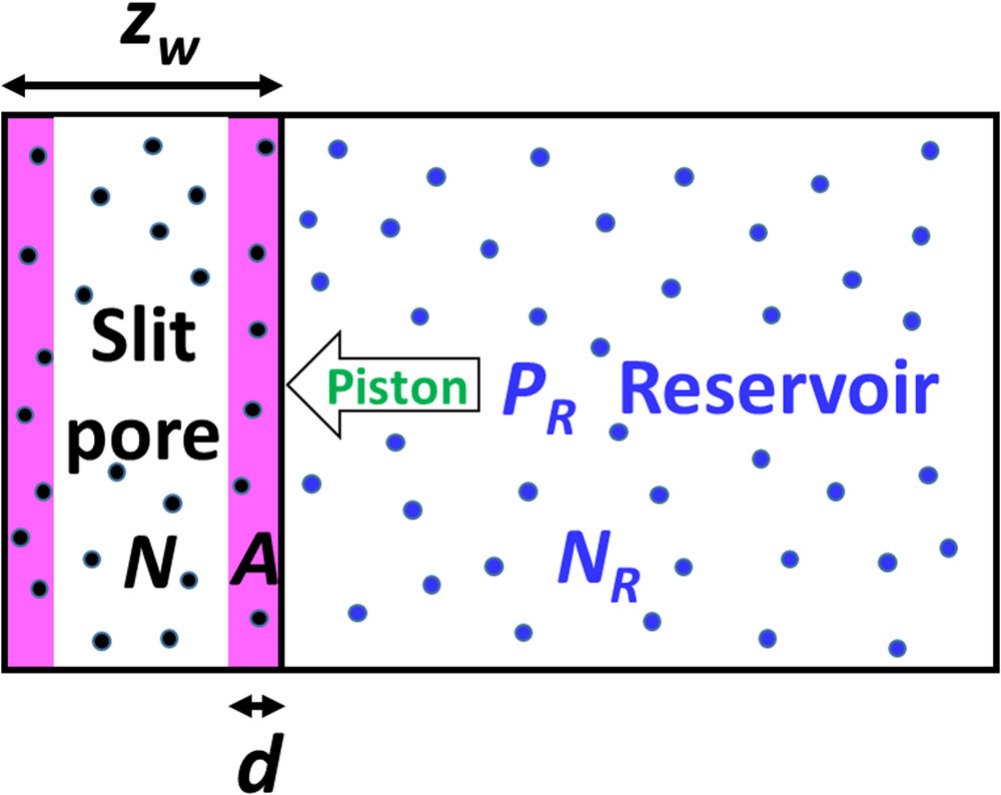
Schematic presentation of the considered system. *N* fluid particles (black dots) are confined in a slit pore connected to a pressure reservoir of *N*_*R*_ particles (blue dots) of the same fluid with a pressure of *P*_*R*_, both at the same temperature. The pore wall of surface area, *A*, on the right can slide frictionlessly and serves as a piston also, thus the pore width ${z}_{w}$ (distance between the two pore walls) fluctuates. The fluid particles in the pink zones interact with the pore walls through a square-well potential of a range *d*.

Using exact statistical-mechanics results, we proceed to check the validity of all the formal thermodynamic results presented above. Confined fluids are chosen for such an illustration since they have many behaviors different from macroscopic ones (see, e.g. refs. ^23–30^, not intending to be an exhaustive bibliography of this vast domain), and provide also an excellent ground for testing nanothermodynamics and for incubating new concepts and new ideas for nanoscale thermodynamics^8–10,19,31^. We consider a fluid confined in a slit pore with a fluctuating width, ${z}_{w}$. The fluid interacts with each pore wall through a square-well potential, i.e.,

26 $${V}_{{fw}}\left({z}_{i}\right)=\phi \left({z}_{i}\right)+\phi \left({z}_{w}-{z}_{i}\right),$$

27 $$\phi \left({z}_{i}\right)=\left\{\begin{array}{cc}{{\infty }},\, & {z}_{i}\, < \,0\\ -\varepsilon,& 0\,\le \,{z}_{i}\,\le \,d\\ 0,& {z}_{i}\ge d\end{array},\right.$$

where ${z}_{i}$ is the *i*th fluid particle’s component of the position vector along the direction perpendicular to the pore walls. The left wall is fixed at $z=0$ and the right fluctuating one at $z={z}_{w}$ serves also as a piston for the *pTN*-ensemble (see Fig. 1). The value of $\varepsilon$ can be positive (attractive fluid-wall interaction) or negative (repulsive fluid-wall interaction). The fluid-fluid interaction is neglected, i.e., an ideal gas. The smallness concerns just the system size in the direction perpendicular to the confining walls. The pore width, i.e., average piston position $\left\langle {z}_{w}\right\rangle$, can be very small while the surface size, i.e., $\sqrt{A}$ can be much larger than $\left\langle {z}_{w}\right\rangle$, (*A*: surface area of one wall). The exact results of statistical-mechanics can be obtained analytically for this model.

**Fig. 2:**
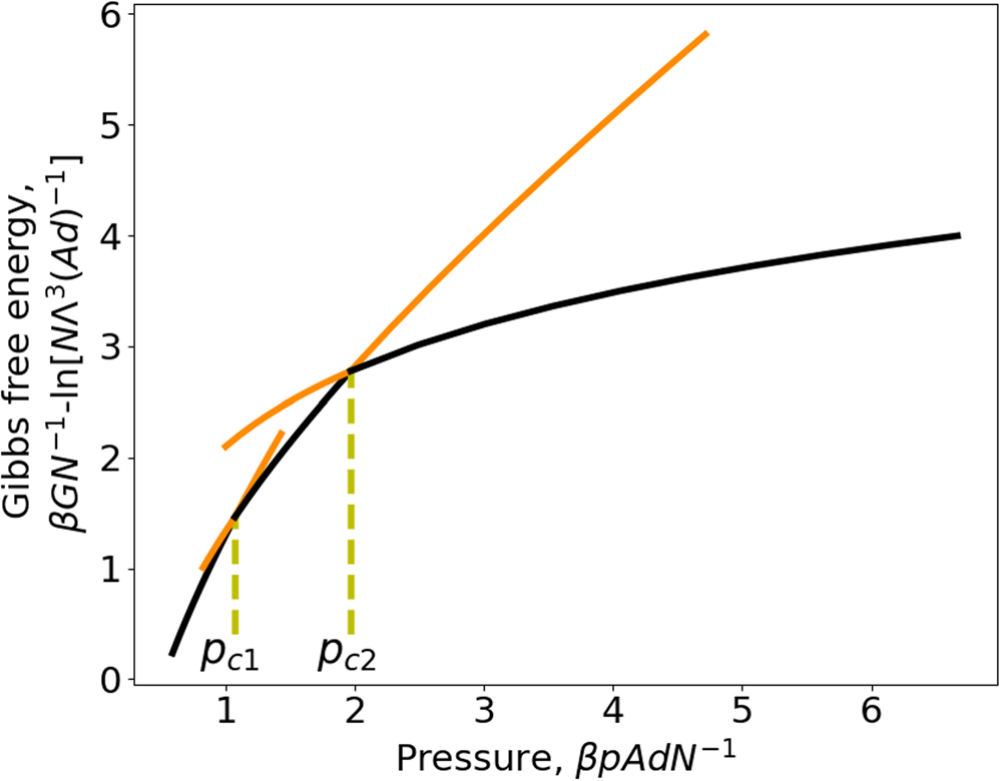
Gibbs free energy as a function of pressure of an ideal gas confined in a slit pore. Repulsive square-well gas-wall potential ($\varepsilon \, < \,0$) at a reduced temperature, $T=0.9525\left|\varepsilon \right|{{k}_{B}}^{-1}\,({{{{{{\rm{e}}}}}}}^{\beta \varepsilon }=0.35)$. The large-pore branch (the left one) and the intermediate-pore branch (the middle one) cross at ${p}_{c1}$, the intermediate-pore branch and the small-pore branch (the right one) cross at ${p}_{c2}$. The black parts represent thermodynamically stable states while the orange parts correspond to metastable states.

Only the main results are presented in this text and computational details are given in Supplementary Information (SI). The exact result for Gibbs free energy is,

28 $$\beta G=\left\{\begin{array}{cc}N{{{{{\rm{ln}}}}}}(\beta p{\varLambda }^{3})-d{{{{{\mathcal{A}}}}}}\beta p\left({{{{{{\rm{e}}}}}}}^{\beta \varepsilon }-1\right),& \left\langle {z}_{w}\right\rangle \ge 2d\\ N{{{{{\rm{ln}}}}}}\frac{{\beta p\varLambda }^{3}}{2-{{{{{{\rm{e}}}}}}}^{\beta \varepsilon }}-\beta \varepsilon N-\frac{d\beta p{{{{{\mathcal{A}}}}}}\left({{{{{{\rm{e}}}}}}}^{\beta \varepsilon }-1\right)}{2-{{{{{{\rm{e}}}}}}}^{\beta \varepsilon }},& d\le \left\langle {z}_{w}\right\rangle \le 2d\\ N{{{{{\rm{ln}}}}}}({\beta p\varLambda }^{3})-2\beta \varepsilon {N},& 0 \, < \,\left\langle {z}_{w}\right\rangle \le d\end{array}\right.$$

where $\varLambda$ is the thermal wavelength, $\beta={\left({k}_{B}T\right)}^{-1}$ (${k}_{B}$: Boltzmann constant), and ${{{{{\mathcal{A}}}}}}=2A$. The first line on the RHS of Eq. (28) is the result for weakly or normally confined situations, i.e., average pore width in the range $\left\langle {z}_{w}\right\rangle > 2d$, the second line for strongly confined situations with $d\le \left\langle {z}_{w}\right\rangle \le 2d$ and the third line for extremely confined situation with $0 \, < \,\left\langle {z}_{w}\right\rangle \le d$. It is straightforward to check that Eq. (28) reduces to the bulk ideal gas result, i.e., $\beta {G}^{{bulk}}=N{{{{{\rm{ln}}}}}}(\beta p{\varLambda }^{3})$, for $\varepsilon=0$ or $T\to \infty$. Despite the simple expression given by Eq. (28), Gibbs free energy for the considered model is a quite complicated function. In fact, Eq. (28) gives not only results for thermodynamically stable states but also for some metastable states. The results for *G* in the case of a repulsive fluid-wall interaction at $T=0.9525\left|\varepsilon \right|{{k}_{B}}^{-1}$ (${{{{{{\rm{e}}}}}}}^{\beta \varepsilon }=0.35$) are presented in Fig. 2. On each branch, we find thermodynamically stable states (black parts) but also some metastable states (orange parts with higher Gibbs free energy than the stable states). So, the Gibbs free energy is not a single-valued function and in some region, it can have even three values for a given pressure (see the region between ${p}_{c1}$ and ${p}_{c2}$ in Fig. 2). The two crossing points, ${p}_{c1}$ and ${p}_{c2}$ are cusps, i.e., the first derivative at these points is discontinuous, which gives, in consequence two discontinuous jumps of pore width. We will see this when discussing the compression-expansion isotherm.

The compression-expansion isotherm, i.e., *p* as a function of $\left\langle {z}_{w}\right\rangle$ at a fixed *T*, is given by,

29 $$\frac{\beta {pAd}}{N}={p}^{*}\left(\left\langle {z}_{w}\right\rangle \right)=\left\{\begin{array}{cc}\frac{1}{\left\langle {z}_{w}\right\rangle /d+2\left({{{{{{\rm{e}}}}}}}^{\beta \varepsilon }-1\right)},& \left\langle {z}_{w}\right\rangle \ge 2d\\ \frac{1}{\left\langle {z}_{w}\right\rangle /d+\frac{2\left({{{{{{\rm{e}}}}}}}^{\beta \varepsilon }-1\right)}{\left(2-{{{{{{\rm{e}}}}}}}^{\beta \varepsilon }\right)}},& d\le \left\langle {z}_{w}\right\rangle \le 2d\\ \frac{1}{\left\langle {z}_{w}\right\rangle /d},\hfill & 0 \, < \, \left\langle {z}_{w}\right\rangle \le d\end{array}\right..$$

**Fig. 3:**
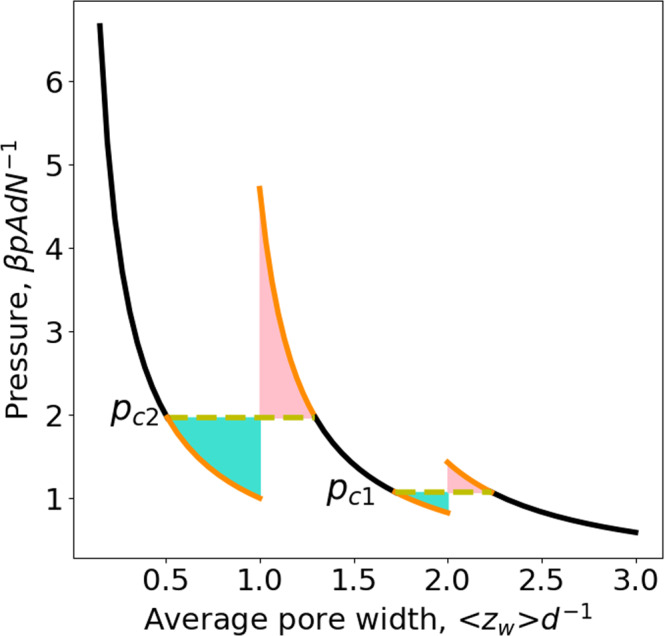
Compression-expansion isotherm of an ideal gas confined in a slit pore. Repulsive square-well gas-wall potential ($\varepsilon \, < \, 0$) at a reduced temperature, $T=0.9525\left|\varepsilon \right|{{k}_{B}}^{-1}\,({{{{{{\rm{e}}}}}}}^{\beta \varepsilon }=0.35)$. The large-pore branch is the right one giving stable states on the black part and metastable states on the orange part (above ${p}_{c1}$). The intermediate-pore branch is the middle one giving stable states on the black part and metastable states on the two orange parts (below ${p}_{c1}$ or above ${p}_{c2}$). The small-pore branch is the left one giving stable states on the black part and metastable states on the orange part (below ${p}_{c2}$). The pink zone (formed by the curve corresponding to over-compressed metastable states and the horizontal line ${p}_{c2}$) has the equal area as the turquoise zone (formed by the curve corresponding to over-expanded metastable states and the horizontal line ${p}_{c2}$). The other pink and turquoise zones formed with ${p}_{c1}$ have also the equal area.

First of all, it is reassuring to see that Eq. (29) reduces to the equation of state of a bulk ideal gas for $\varepsilon=0,\,{{{{{\rm{i}}}}}}.{{{{{\rm{e}}}}}}.,\,p={k}_{B}{TN}{\left(A\left\langle {z}_{w}\right\rangle \right)}^{-1}$. Figure 3 gives an illustration for $T=0.9525\left|\varepsilon \right|{{k}_{B}}^{-1}\,({{{{{{\rm{e}}}}}}}^{\beta \varepsilon }=0.35)$. The two discontinuous jumps of pore width at ${p}_{c1}$ and ${p}_{c2}$ correspond precisely to the discontinuous derivative of Gibbs free energy at the two crossing points in Fig. 2, which are cusps. It is very interesting to note that drawing a horizontal line corresponding to ${p}_{c1}$ (or to ${p}_{c2}$) in Fig. 3 (yellow dashed lines) provides a kind of Maxwell construction. It can be shown analytically that the turquoise and pink zones have the equal area (see Supplementary Discussion 3 for more details). So, the results presented in Figs. 2 and 3 seem to indicate an underlying phase transition although an ideal gas is considered here.

Despite the striking similarity of the results of Figs. 2 and 3 to those for two successive first-order phase transitions, we will show now there is no underlying phase transition for the considered system. From Eq. (28), we obtain respectively the differential chemical potential,

30 $$\beta \mu={\left[\frac{\partial \left(\beta G\right)}{\partial N}\right]}_{T,\,p,\,{{{{{\mathcal{A}}}}}}}=\left\{\begin{array}{cc}{{{{{\rm{ln}}}}}}\left(\beta p{\varLambda }^{3}\right)\,,\, \hfill & 2d\le \left\langle {z}_{w}\right\rangle \\ {{{{{\rm{ln}}}}}}\frac{{\beta p\varLambda }^{3}}{2-{{{{{{\rm{e}}}}}}}^{\beta \varepsilon }}-\beta \varepsilon \,\hfill & d\le \left\langle {z}_{w}\right\rangle \le 2d\\ {{{{{\rm{ln}}}}}}\left({\beta p\varLambda }^{3}\right)-2\beta \varepsilon,& 0 \, < \, \left\langle {z}_{w}\right\rangle \, \le \,d\end{array},\right.$$

and the integral chemical potential,

31 $$\beta \hat{\mu }=\frac{\beta G}{N}=\left\{\begin{array}{cc}{{{{{\rm{ln}}}}}}\left(\beta p{\varLambda }^{3}\right)-\frac{d{{{{{\mathcal{A}}}}}}\beta p\left({{{{{{\rm{e}}}}}}}^{\beta \varepsilon }-1\right)}{N},\hfill & 2d\le \left\langle {z}_{w}\right\rangle \\ {{{{{\rm{ln}}}}}}\frac{{\beta p\varLambda }^{3}}{2-{{{{{{\rm{e}}}}}}}^{\beta \varepsilon }}-\beta \varepsilon -\frac{d\beta p{{{{{\mathcal{A}}}}}}\left({{{{{{\rm{e}}}}}}}^{\beta \varepsilon }-1\right)}{N\left(2-{{{{{{\rm{e}}}}}}}^{\beta \varepsilon }\right)},& d\le \left\langle {z}_{w}\right\rangle \le 2d\\ {{{{{\rm{ln}}}}}}\left({\beta p\varLambda }^{3}\right)-2\beta \varepsilon,\hfill & 0 \, < \, \left\langle {z}_{w}\right\rangle \le d\end{array}.\right.$$

Equations (30) and (31) show distinct differential and integral chemical potentials for the large-pore and intermediate-pore ranges while for the small-pore range $\mu=\hat{\mu }$. Equation (30) shows for the weakly and normally confined situations, the confined and bulk fluids have the same chemical potential, i.e., $\beta \mu=\beta {\mu }^{{bulk}}={{{{{\rm{ln}}}}}}(\beta P{\varLambda }^{3})$ while a disjoining chemical potential is found in the strongly and extremely confined situations, i.e.,

32 $$\beta \varpi=\beta \left(\mu -\,{\mu }^{{bulk}}\right)=\left\{\begin{array}{cc}0\,,\hfill & 2d\le \left\langle {z}_{w}\right\rangle \\ -\beta \varepsilon -{{{{{\rm{ln}}}}}}\left(2-{{{{{{\rm{e}}}}}}}^{\beta \varepsilon }\right)\,,& \,d\le \left\langle {z}_{w}\right\rangle \le 2d\\ -2\beta \varepsilon,\,,\hfill & 0 \, < \, \left\langle {z}_{w}\right\rangle \le d\end{array}.\right.$$

**Fig. 4:**
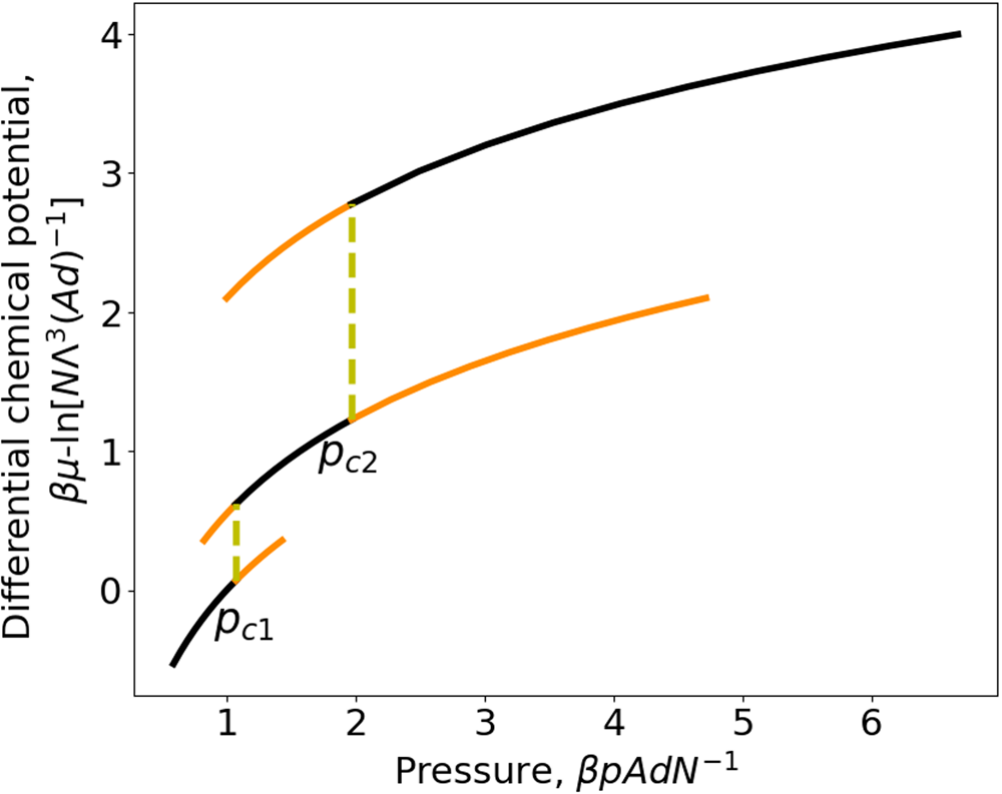
Differential chemical potential as a function of pressure of an ideal gas confined in a slit pore. Repulsive square-well gas-wall potential $(\varepsilon \, < \, 0)$ at a reduced temperature, $T=0.9525\left|\varepsilon \right|{{k}_{B}}^{-1}\,({{{{{{\rm{e}}}}}}}^{\beta \varepsilon }=0.35)$. The large-pore branch is the lowest one giving thermodynamically stable states on the black part and metastable states on the orange part (above ${p}_{c1}$). The intermediate-pore branch is the middle one giving thermodynamically stable states on the black part and metastable states on the two orange parts (below ${p}_{c1}$ or above ${p}_{c2}$). The small-pore branch is the highest one giving stable states on the black part and metastable states on the orange part (below ${p}_{c2}$).

This corroborates our initial guess that a disjoining chemical potential can arise in a *pTN*-ensemble. Moreover, it is to note that *ϖ* is only a function of *T* but independent of *p*. The results for the differential chemical potential given by Eq. (30) are presented in Fig. 4. From Fig. 4, we see immediately that the chemical potential changes discontinuously at ${p}_{c1}$ and ${p}_{c2}$. The first jump at ${p}_{c1}$ is precisely the disjoining chemical potential for the strongly confined situation (i.e., states on the middle black curve). The sum of the two jumps gives the disjoining chemical potential for the extremely confined situation (i.e., states on the black part of the highest branch). Now, it becomes clear that none of the horizontal yellow lines in Fig. 3 corresponds to an underlying phase transition since the two end points do not have the same chemical potential. So, the removal of the metastable states from the results given in Figs. 2–4 can be only qualified as a quasi Maxwell construction since it does not lead to equal chemical potential while the original one for first-order phase transitions does. Nevertheless, this quasi Maxwell construction allows for separating thermodynamically stable states from the metastable ones. Moreover, a first-order phase transition can only take place below some critical temperature while the discontinuous jumps shown in Fig. 3 subsist at any temperature $T \, < \, \infty$. With the increase of the temperature, the amplitude of the discontinuous jumps diminishes. But, they disappear completely only when $T\to \infty$, in this limit the three branches in Fig. 3 reduce to a smooth curve and the result of the bulk ideal gas is recovered. This is another reason why the discontinuous jumps seen in Fig. 3 should not be interpreted as a phase transition.

**Fig. 5:**
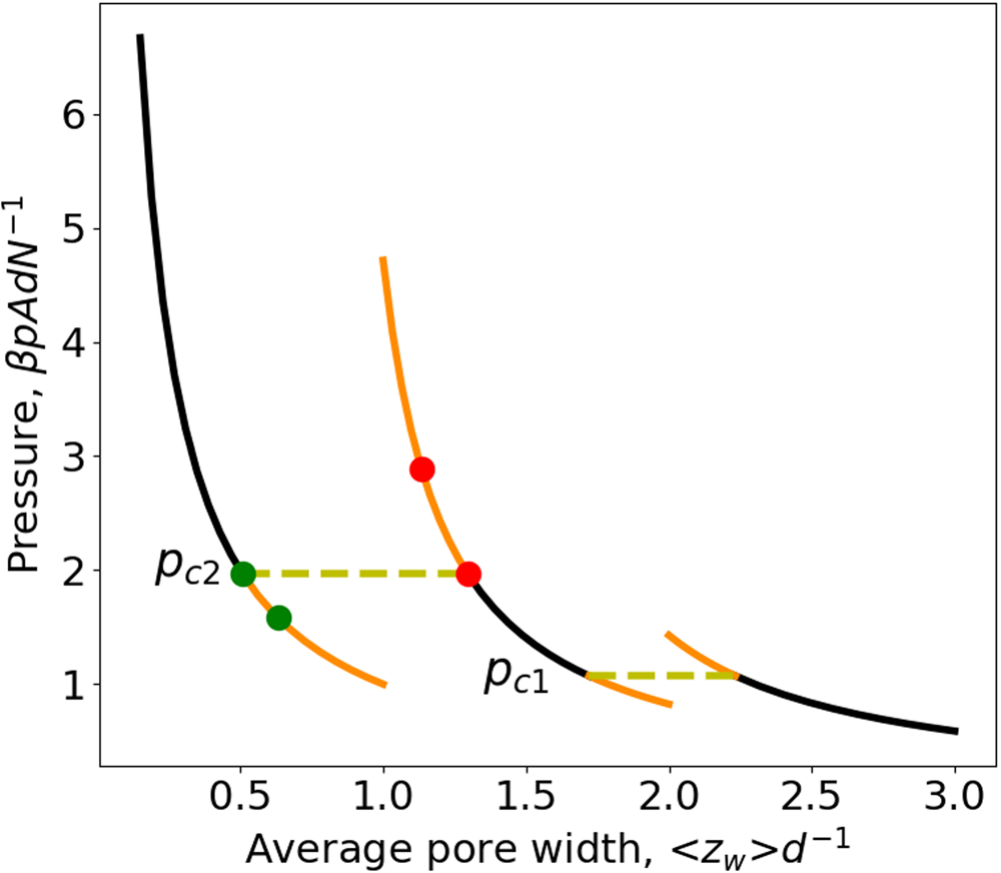
Isobaric-isothermal Monte-Carlo simulation results. Evidence for the hysteresis in the compression-expansion isotherm for an ideal gas confined in a slit pore (computational conditions and details are given below in Methods section). Repulsive square-well gas-wall potential ($\varepsilon \, < \, 0$) at a reduced temperature, $T=0.9525\left|\varepsilon \right|{{k}_{B}}^{-1}\,({e}^{\beta \varepsilon }=0.35)$. *pTN*-ensemble Monte-Carlo simulation results: red dots (obtained in a compression process), green dots (obtained in an expansion process); Exact analytical result given by Eq. (29): lines (the same results as those shown in Fig. 3).

It is well-known that the metastable states associated with a first-order phase transition lead to hysteresis in the compression-expansion isotherm. Figure 3 shows that metastable states are also found near the discontinuous jumps. Although they do not correspond to any underlying phase transition, we can wonder whether they lead also to some hysteresis, e.g., over-compressed states during a compression process or over-expanded states during an expansion process. To answer this question, we performed some *pTN*-ensemble Monte-Carlo simulations (computational conditions and details are given below in Methods section). The simulation results presented in Fig. 5 show that the over-compressed and over-expanded metastable states can be indeed observed. When the pressure of the reservoir is fixed at ${p}_{c2}$ and the simulation is started with a pore width in the range $d\le \left\langle {z}_{w}\right\rangle \le 2d$, the piston does not jump to the pore-width range $0 \, < \, \left\langle {z}_{w}\right\rangle \le d$ and the obtained result is given by the red dot at the right end point of the horizontal line $p={p}_{c2}$. At the same pressure, if the simulation is started in the pore-width range $0 \, < \, \left\langle {z}_{w}\right\rangle \le d$, no piston jump is observed either and the obtained result is given by the green dot at the left end point of the horizontal line. No spontaneous jumps are due to the high free energy barrier to be overcome for such jumps. Such barriers make it possible to observe also over-compressed states, e.g., the other red point in Fig. 5 and over-expanded states, e.g., the other green point in Fig. 5.

The differential surface tension is given by,

33 $$\beta \gamma={\left[\frac{\partial \left(\beta G\right)}{\partial {{{{{\mathcal{A}}}}}}}\right]}_{T,p,N}=\left\{\begin{array}{cc}-d\beta p\left({e}^{\beta \varepsilon }-1\right),\hfill & 2d\le \left\langle {z}_{w}\right\rangle \\ -\frac{d\beta p\left({e}^{\beta \varepsilon }-1\right)}{2-{e}^{\beta \varepsilon }},\, \hfill & d\le \left\langle {z}_{w}\right\rangle \le \,2d\\ 0,\hfill & 0 \, < \, \left\langle {z}_{w}\right\rangle \le d\end{array}.\right.$$

**Fig. 6:**
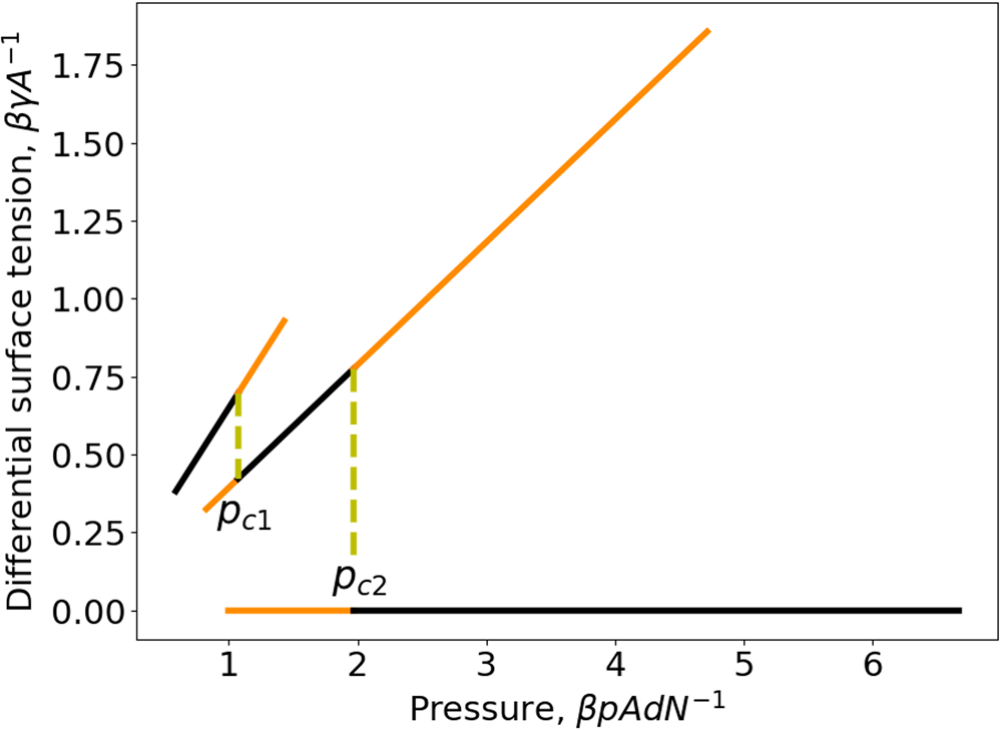
Differential surface tension as a function of pressure of an ideal gas confined in a slit pore. Repulsive square-well gas-wall potential $(\varepsilon < 0)$ at a reduced temperature, $T=0.9525\left|\varepsilon \right|{{k}_{B}}^{-1}\,({{{{{{\rm{e}}}}}}}^{\beta \varepsilon }=0.35)$. The large-pore branch is the left one with the thermodynamically stable states on the black part and metastable states on the orange part $(p \, > \, {p}_{c1})$. The intermediate-pore branch is the middle one with the thermodynamically stable states on the black part and metastable states on the two orange parts ($p \, < \, {p}_{c1}\,{{{{{\rm{or}}}}}}\,p \, > \, {p}_{c2}$). The small-pore branch is the right one with the thermodynamically stable states on the black part and metastable state on the orange part $(p \, < \, {p}_{c2})$.

When *p* in Eq. (33) is replaced by *µ* with the help of Eq. (30), we recover the result for *γ* obtained with grand potential in Ref. ^19^. This evidences the ensemble-independence of differential surface tension, thus justifies its denotation without index indicating the used thermodynamic potential to define it. Equations (30) and (33) show also *µ* and *γ* depend only on *T* and *p* but not on $\hat{\varphi }=N{{{{{{\mathcal{A}}}}}}}^{-1}$ for the considered model. The results for the differential surface tension given by Eq. (33) are presented in Fig. 6. In the range of small pore width $0 \, < \, \left\langle {z}_{w}\right\rangle \le d$, the differential surface tension vanishes since the confined ideal gas becomes a homogeneous one.

We also used the mechanical definition to calculate surface tension (see SI for details), i.e.,

34 $${\gamma }^{{Me}{ch}}=\frac{1}{2}{\int }_{0}^{L}{dz}\left[{p}_{N}\left(z\right)-{p}_{T}\left(z\right)\right],$$

where ${p}_{N}\left(z\right)$, ${p}_{T}\left(z\right)$ are respectively the normal and transverse components of pressure tensor and the factor $1/2$ accounts for the two interfaces. The normal component of the pressure tensor is constant everywhere and equal to *p* to assure the mechanical equilibrium (see SI for details). The mechanical definition gives the differential surface tension as in a *µTV*-ensemble^19^.

Integral surface tension is given by,

35 $$\beta {\hat{\gamma }}_{G}=\frac{\beta \left(G-{\mu }^{{bulk}}N\right)}{{{{{{\mathcal{A}}}}}}}=\left\{\begin{array}{cc}-d\beta p\left({{{{{{\rm{e}}}}}}}^{\beta \varepsilon }-1\right),\, \hfill & 2d\le \left\langle {z}_{w}\right\rangle \\ -\frac{d\beta p\left({{{{{{\rm{e}}}}}}}^{\beta \varepsilon }-1\right)}{2-{{{{{{\rm{e}}}}}}}^{\beta \varepsilon }}+\frac{\beta \varpi N}{{{{{{\mathcal{A}}}}}}},& d\le \left\langle {z}_{w}\right\rangle \le 2d\\ \frac{\beta \varpi N}{{{{{{\mathcal{A}}}}}}},\, \hfill & 0 \, < \, \left\langle {z}_{w}\right\rangle \le d\end{array}.\right.$$

Equations (33) and (35) show for weakly and normally confined situations, ${\hat{\gamma }}_{G}=\gamma$, while ${\hat{\gamma }}_{G}\, \ne \, \gamma$ for strongly and extremely confined fluids. Now, we show for strongly confined situation, the integral surface tension calculated in *pTN*-ensemble is not equal to that from a *µTN*-ensemble, i.e., ${\hat{\gamma }}_{G}\, \ne \, {\hat{\gamma }}_{\Omega }$. Expressing *N* and *p* in terms of *µ* and *V* on the second line of Eq. (35) (see section 1.2.4 of Supplementary Note 1.2 for more details), we obtain,

36 $$\beta {\hat{\gamma }}_{G}\left(T,\, \mu,\, V{{{{{\mathscr{,\, }}}}}}{{{{{\mathcal{A}}}}}}\right)=	-\frac{{V{{{{{\rm{e}}}}}}}^{\beta \left(\mu+\varepsilon \right)}}{{{{{{\mathcal{A}}}}}}{\varLambda }^{3}}\left({{{{{{\rm{e}}}}}}}^{\beta \varepsilon }-1\right)\left[\frac{d{{{{{\mathcal{A}}}}}}}{V}\left(1-\beta \varpi \right)-\beta \varpi \frac{\left(2-{{{{{{\rm{e}}}}}}}^{\beta \varepsilon }\right)}{{{{{{{\rm{e}}}}}}}^{\beta \varepsilon }-1}\right],\, \\ 	d\le \left\langle {z}_{w}\right\rangle \le 2d.$$

This is indeed different from the result obtained with a grand potential^19^, i.e.,

37 $$\beta {\hat{\gamma }}_{\Omega }\left(T,\,\mu,\,V{{{{{\mathscr{,}}}}}}\,{{{{{\mathcal{A}}}}}}\right)=-\frac{V}{{{{{{\mathcal{A}}}}}}}\frac{{{{{{{\rm{e}}}}}}}^{\beta \mu }}{{\varLambda }^{3}}\left({{{{{{\rm{e}}}}}}}^{\beta \varepsilon }-1\right)\left[1+\left(\frac{d{{{{{\mathcal{A}}}}}}}{V}-1\right){{{{{{\rm{e}}}}}}}^{\beta \varepsilon }\right]\,,\,d\le \left\langle {z}_{w}\right\rangle \le 2d.$$

Thus, the ensemble-dependence of integral surface tensions is evidenced. Nevertheless, ${\hat{\gamma }}_{\Omega }$ and ${\hat{\gamma }}_{G}$ are related through the following relation (see section 1.2.4 of Supplementary Note 1.2 for the check of its validity),

38 $${\hat{\gamma }}_{\Omega }={\hat{\gamma }}_{G}-\left(\varpi \hat{\varphi }+\Pi \hat{{{{{{\mathscr{l}}}}}}}\right).$$

Here, we make a special warning for the calculation of surface tension by molecular simulations. Various methods have been developed, some based on its integral definition (e.g., extracting surface contribution from grand potential or integrating Gibbs adsorption equation) and other ones resorting to its differential definition (e.g., mechanical definition). They all give the same result for weakly or normally confined situations. However, for strongly and extremely confined situations, it is indispensable to know that integral and differential surface tensions are different to avoid any confusion and to interpret correctly the obtained results.

The integral adsorption-induced strain is given by,

39 $$\hat{\Delta }=\left\{\begin{array}{cc}-d\left({{{{{{\rm{e}}}}}}}^{\beta \varepsilon }-1\right),& \,2d \, < \, \left\langle {z}_{w}\right\rangle \\ -\frac{d\left({{{{{{\rm{e}}}}}}}^{\beta \varepsilon }-1\right)}{2-{{{{{{\rm{e}}}}}}}^{\beta \varepsilon }}\,,& d \, < \, \left\langle {z}_{w}\right\rangle \, < \,2d\\ 0 \hfill & 0 \, < \, \left\langle {z}_{w}\right\rangle \le d\end{array}.\right.$$

Since $\hat{\Delta }$ does not depend on $\hat{\varphi }$, we obtain immediately that $\hat{\Delta }=\Delta$. In fact, this equality is due to the independence of disjoining chemical potential on pressure. Subtracting bulk Gibbs-Duhem equation from Eq. (8), we obtain,

40 $$\left(S-{S}^{{bulk}}\right){dT}-\left(V-{V}^{{bulk}}\right){dp}+{Nd}\varpi {{{{{\mathscr{+}}}}}}{{{{{\mathcal{A}}}}}}d\gamma=0,$$

which gives immediately,

41 $${\left(\frac{\partial \gamma }{\partial p}\right)}_{T}=\hat{\Delta }-\hat{\varphi }{\left(\frac{\partial \varpi }{\partial p}\right)}_{T}=\hat{\Delta }.$$

The second equality on the RHS of Eq. (41) is obtained when

42 $${\left(\frac{\partial \varpi }{\partial p}\right)}_{T}=0.$$

Comparing Eq. (41) with Eq. (25) shows immediately $\hat{\Delta }=\Delta$. Since we have not succeeded in proving Eq. (42) in general, we consider it as a fortuitous result for the considered model. From Eqs. (35) and (39), we can check readily that the adsorption equation for ${\hat{\gamma }}_{G}$ given in Eq. (21) holds.

## Discussion

More than eighty years after the discovery of disjoining pressure, we reveal that disjoining chemical potential can also arise for thin slice-shape systems. Disjoining pressure links differential surface tension to the integral surface tension defined with grand potential. In parallel, disjoining chemical potential links differential surface tension to the integral surface tension defined with Gibbs free energy. As environment dependence is concerned, intensive thermodynamic variables can be classified into two categories. The differential intensive variables are ensemble-independent while the integral intensive variables are ensemble-dependent. For example, integral pressure and disjoining pressure arise in a grand canonical ensemble while integral chemical potential and disjoining chemical potential arise in a *pTN*-ensemble but not in a grand canonical ensemble. Acquaintance of this dualism of environment dependence is indispensable for understanding the behaviors of small systems and for interpreting correctly the observations from experiments or simulations for such systems. For example, only when the concepts of differential and integral surface tensions and the ensemble dependence of the latter are taken into full account, can one understand the possible different results obtained from various simulations based on different definitions of surface tension. The results presented in this article allow for relating these results to each other and also for checking their thermodynamic consistency. Moreover, only differential intensive variables can be used for Legendre transforms to change correctly one thermodynamic potential to another.

Despite its apparent simplicity, the model used for illustration, i.e., an ideal gas confined in a slit pore, gives some complicated behavior. The exact analytical statistical-mechanics results involve both thermodynamically stable and metastable states. The discontinuous jumps in the compression-expansion isotherm cannot be attributed to any underlying phase transition since the two end points of such jumps do not have the same chemical potential. Moreover, the discontinuous jumps subsist at any temperature $T \, < \, \infty$ while a first-order phase transition has a critical point at a finite temperature. The metastable states are observable in *pTN*-ensemble Monte-Carlo simulations, which lead to hysteresis in the compression-expansion isotherm. So, such a hysteresis alone can no longer be taken as the signature of a phase transition and this complicates the investigation of phase transitions in small systems. It becomes mandatory to check the equality of *µ* and that of *p* between the two coexisting phases. Integral surface tension, both ${\hat{\gamma }}_{\Omega }$ and ${\hat{\gamma }}_{G}$, are in principle experimentally measurable. The generalized adsorption equations derived recently^19^ and in the present work relate them respectively to integral adsorption and adsorption-strain. The adsorption isotherms can be obtained in routine laboratory measurements We expect our present work will give some impetus for elaborating experimental measurements of adsorption-strain with deformable porous materials. Further investigations with more realistic models by molecular simulations will contribute to the general validation of our findings.

## Methods

### Analytical statistical-mechanics calculations

The general thermodynamics formalism might appear abstract. A concrete illustration helps substantiating concepts like disjoining chemical potential, ensemble-dependence of integral surface tensions. For such an illustration, some exact statistical-mechanics calculations are carried out analytically for a well-defined simple model by neglecting interaction between fluid particles. Such a simplification does not affect the generality of the illustration since the very existence of disjoining chemical potential, the ensemble-dependence of the integral surface tensions and the validity of the proposed adsorption equations are all clearly revealed. So, the chosen model fulfills perfectly its role for illustration. Moreover, the obtained analytic results enforce its demonstrative power. Interparticle interaction gives certainly an additional contribution to both differential and integral surface tensions, but it cannot cancel the main effect responsible for the two distinct surface tensions, which is due to particle-wall interaction. We believe the interaction between fluid particles even enhances the fluid-mediated force between pore walls. Moreover, the interparticle interaction should not affect the existence of the observed compression-expansion hysteresis since it is mainly due to particle-wall interaction. Since all our formal results are derived from thermodynamics, they hold for any system whatever are the interactions between its components.

### Isobaric-isothermal Monte-Carlo simulation

The standard *pTN*-ensemble Monte-Carlo method^32^ can be readily adapted for the model considered here. The MC simulations are performed under the following computational conditions: (1) reduced temperature: $T=0.9525\left|\varepsilon \right|{{k}_{B}}^{-1}$; (2) number of particles: $N=432$; (3) square-shape pore wall of side length equal to 12*d*; (4) number of MC cycles for preparing the system to equilibrium: 4500; (5) number of production MC cycles to obtain the simulation results given in Fig. 5: 9000. The statistical errors of the Monte-Carlo simulations do not exceed 0.5% (error bar smaller than the dot size in Fig. 5).

## Supplementary information

Supplementary information

## Data Availability

The author declares that the data supporting the findings of this study are available in the paper and its Supplementary Information files. All relevant data are available from the corresponding author on request.

## Code availability

The isobaric-isothermal Monte-Carlo simulation code used to obtain the simulation results presented in Fig. 5 is available from the corresponding author on request.
