# Supplementary material for: Nanoscale thermodynamics needs the concept of a disjoining chemical potential
Source: Nat Commun. 2023 Apr 1;14:1824. doi: 10.1038/s41467-023-36970-7 (PMC10067931; doi:10.1038/s41467-023-36970-7)
Supplement: Supplementary file 1 — Supplementary information [file 41467_2023_36970_MOESM1_ESM.pdf]

Supplementary information

Nanoscale thermodynamics needs the concept of a disjoining chemical potential

W. Dong *

* Corresponding author. Email: wei.dong@ens-lyon.fr

Supplementary discussion 1. Exact *pTN*-ensemble statistical-mechanics results for an ideal gas in a slit-pore

The statistical-mechanics calculations presented in this Supplementary Information are carried out for an ideal gas confined in a slit pore connected to a pressure reservoir treated on an equal footing of the confined gas, i.e., an explicit reservoir with N_R particles (see Figure 1 in the main text). The whole composed system is in a canonical ensemble at temperature, T, with N_T particles ($N_T = N + N_R$) in a total volume of V_T. The interaction potential between a fluid particle and the two pore walls is given by,

$$V_{fw}(z_i) = \phi(z_i) + \phi(z_w - z_i), \tag{1.1}$$

$$\phi(z_i) = \begin{cases} \infty & , \quad z_i < 0 \\ -\varepsilon & , \quad 0 < z_i < d \\ 0 & , \quad z_i > d \end{cases} . \tag{1.2}$$

The left wall is located at $z = 0$ (fixed) and the right one at $z = z_w$ can fluctuate. The value of ε can be positive (attractive fluid-solid interaction) or negative (repulsive fluid-solid interaction). The fluid-fluid interaction is neglected, i.e., an ideal gas being considered.

Depending on the value of averaged pore width, $\langle z_w \rangle$, we consider separately different situations: i) weakly and normally confined situations, i.e., $\langle z_w \rangle \geq 2d$ (d : fluid-wall interaction range, see Eq. (1.2) and Fig. 1 in the main text); ii) strongly confined situation, i.e., $d \leq \langle z_w \rangle \leq 2d$; iii) extremely confined situation, i.e., $0 < \langle z_w \rangle \leq d$.

Supplementary note 1.1. Weakly and normally confined situations ($\langle z_w \rangle \geq 2d$)

1.1.1. Total partition function and Gibbs free energy of confined fluid ($\langle z_w \rangle \geq 2d$)

With the composed system depicted in Fig. 1 of the main text, we determine first the partition function

of the whole system from which Gibbs free energy of the confined fluid can be obtained. Since the composed system is in a canonical ensemble, its partition function is given by,

$$Z_T = \frac{1}{\Lambda^{3N}\Lambda^{3N_R}\Lambda_w N! N_R!}\int_{2d}^{L_T} dz_w \left\{A\int_0^{z_w} dz\, exp\left[-\beta V_{fw}(z)\right]\right\}^N \left(A\int_{z_w}^{L_T} dz\right)^{N_R}, \tag{1.3}$$

where Λ and Λ_W are respectively the thermal wavelengths of fluid particles and the fluctuating wall (piston), A surface area of one wall, L_T width of the total system. In order to deal with only unitless quantities, we reduce all the length related quantity by the fluid thermal wavelength and the reduced quantities are denoted with a star. Thus, Eq. (1.3) becomes,

$$\begin{aligned} Z_T &= \frac{A^{*N_T}}{N! N_R!}\frac{\Lambda}{\Lambda_w}\int_{2d^*}^{L_T^*} dz_w^*\left[z_w^* + 2d^*\left(e^{\beta\varepsilon}-1\right)\right]^N (L_T^* - z_w^*)^{N_R} \\ &= \frac{A^{*N_T} L_T^{*N_R}}{N! N_R!}\frac{\Lambda}{\Lambda_w}\int_{2d^*}^{L_T^*} dz_w^*\left[z_w^* + 2d^*\left(e^{\beta\varepsilon}-1\right)\right]^N \left(1-\frac{z_w^*}{L_T^*}\right)^{N_R} \ . \end{aligned} \tag{1.4}$$

Now, the last factor of the integrant is rewritten as follows,

$$\left(1-\frac{z_w^*}{L_T^*}\right)^{N_R} = \left(1-\frac{N_R A^* z_w^*}{A^* L_T^* N_R}\right)^{N_R} = \left(1-\frac{\beta P_R \Lambda^3 A^* z_w^*}{N_R}\right)^{N_R} = exp(-\beta P_R \Lambda^3 A^* z_w^*) \ . \tag{1.5}$$

When going to the before-last equality in Eq. (1.5), the equation of state of the ideal-gas in the reservoir is used, i.e., $\beta P_R = N_R/(AL_R) = N_R/(AL_T)$, since $L_R \approx L_T$ when $\langle z_w \rangle \ll L_T$. The last equality in Eq. (1.5) is obtained in the thermodynamic limit, i.e., $N_R \to \infty$, $V_R = AL_R \to \infty$ with $N_R/(AL_R)$ remaining finite. Substituting Eq. (1.5) into Eq. (1.4), we obtain,

$$\begin{aligned} Z_T &= \frac{A^{*N_T} L_T^{*N_R}}{N! N_R!}\frac{\Lambda}{\Lambda_w}\int_{2d^*}^{L_T^*} dz_w^*\left[z_w^* + 2d^*\left(e^{\beta\varepsilon}-1\right)\right]^N exp(-\beta P_R \Lambda^3 A^* z_w^*) \\ &= \frac{A^{*N_T} L_T^{*N_R}}{N! N_R!}\frac{\Lambda}{\Lambda_w}\int_{2d^*}^{L_T^*} dz_w^* exp[-\mathcal{H}(z_w^*)] \ , \end{aligned} \tag{1.6}$$

where

$$\mathcal{H}(z_w^*) = \beta P_R \Lambda^3 A^* z_w^* - N ln\left[z_w^* + 2d^*\left(e^{\beta\varepsilon}-1\right)\right]. \tag{1.7}$$

Now, if N scales as A^* and they are very large, the integral in Eq. (1.6) can be calculated by using Laplace method[32] when $\mathcal{H}(z_w^*)$ has a maximum in the integration domain.

$$\mathcal{H}'(z_w^*) = \beta P_R \Lambda^3 A^* - \frac{N}{z_w^* + 2d^*\left(e^{\beta\varepsilon}-1\right)} \ . \tag{1.8}$$

$$\mathcal{H}''(z_w^*) = \frac{N}{\left[z_w^* + 2d^*\left(e^{\beta\varepsilon}-1\right)\right]^2} \ . \tag{1.9}$$

The first and second derivatives given in Eq. (1.8) and Eq. (1.9) show that $\mathcal{H}(z_w^*)$ has indeed a minimum at $\bar{z}_w^*$, i.e.,

$$\bar{z}_w^* = \frac{N}{\beta P \Lambda^3 A^*} - 2d^*\left(e^{\beta\varepsilon}-1\right) \ , \tag{1.10}$$

$$\mathcal{H}(\bar{z}_w^*) = N - N ln\frac{N}{\beta P_R \Lambda^3 A^*} - 2d^*\beta P_R \Lambda^3 A^*\left(e^{\beta\varepsilon} - 1\right) \ . \tag{1.11}$$

Expanding $\mathcal{H}(z_w^*)$ around $\bar{z}_w^*$ in a Taylor series to the second order, we obtain,

$$\mathcal{H}(z_w^*) = \mathcal{H}(\bar{z}_w^*) + \frac{1}{2}\mathcal{H}''(\bar{z}_w^*)(z_w^* - \bar{z}_w^*)^2. \tag{1.12}$$

Substituting Eq (1.12) into Eq (1.6), we obtain,

$$Z_T = \frac{A^{*N_T} L_T^{*N_R}}{N! N_R!} exp[-\mathcal{H}(\bar{z}_w^*)] \frac{\Lambda}{\Lambda_w} \int_{2d^*}^{L_T^*} dz_w^* exp\left[-\frac{1}{2}\mathcal{H}''(\bar{z}_w^*)(z_w^* - \bar{z}_w^*)^2\right] \ , \tag{1.13}$$

In the limits, $N \to \infty$ and $L_T \to \infty$, the integral on the RHS of Eq (1.13) becomes a Gaussian one and we obtain finally,

$$Z_T = \frac{A^{*N_T} L_T^{*N_R}}{N! N_R!} exp[-\mathcal{H}(\bar{z}_w^*)] \frac{\Lambda}{\Lambda_w} \sqrt{\frac{2\pi}{\mathcal{H}''(\bar{z}_w^*)}}$$

$$= \frac{A^{*N_T} L_R^{*N_R}\left(1 + \frac{\langle z_w^* \rangle}{L_R^*}\right)^{N_R}}{N! N_R!} exp[-\mathcal{H}(\bar{z}_w^*)] \frac{\Lambda}{\Lambda_w} \sqrt{\frac{2\pi}{\mathcal{H}''(\bar{z}_w^*)}}$$

$$= Z_R \frac{A^{*N}}{N!} exp[-\mathcal{H}(\bar{z}_w^*) + \beta P_R \Lambda^3 \langle z_w^* \rangle A^*] \frac{\Lambda}{\Lambda_w} \sqrt{\frac{2\pi}{\mathcal{H}''(\bar{z}_w^*)}} \ , \tag{1.14}$$

where $\langle z_w^* \rangle$ is the average width of the slit pore (to be calculated in the next subsection) and Z_R is the partition function of the reservoir, i.e.,

$$Z_R = \frac{A^{N_R} L_R^{N_R}}{\Lambda^{3N_R} N_R!}. \tag{1.15}$$

When going to the last equality of Eq (1.14), the thermodynamic limit is taken, i.e., $N_R \to \infty$ and $(AL_R) \to \infty$ with $N_R/(AL_R) = \beta P_R$ remaining finite. Now, we obtain the Gibbs free energy of the confined fluid by subtracting the Gibbs free energy of the reservoir from that of the total system, i.e.,

$$\beta G(T, P, N, \mathcal{A}) = \beta(G_T - G_R) = \beta(F_T - F_R + P\langle z_w \rangle A) = -ln\frac{Z_T}{Z_R} + \beta P \langle z_w \rangle A$$

$$= N ln(\beta P_R \Lambda^3) - 2d\beta P_R A\left(e^{\beta\varepsilon} - 1\right) \ . \tag{1.16}$$

The term involving $\mathcal{H}''(\bar{z}_w^*)$ becomes negligible in the thermodynamic limit, $N \to \infty$ and $A \to \infty$, thus it does not contribute to the Gibbs free energy.

1.1.2. Average pore width and adsorption strain ($\langle z_w \rangle > 2d$)

The average pore width, $\langle z_w^* \rangle$ can be obtained by first carrying out the easier calculation of $\langle z_w^* + 2d^*\left(e^{\beta\varepsilon} - 1\right) \rangle$, i.e.,

$$\langle z_w^* + 2d^*\left(e^{\beta\varepsilon} - 1\right) \rangle = \frac{A^{*N_T} L_T^{*N_R}}{N! N_R! Z_T} \frac{\Lambda}{\Lambda_w} \int_{2d^*}^{L_T^*} dz_w^* \left[z_w^* + 2d^*\left(e^{\beta\varepsilon} - 1\right)\right]^{N+1} exp(-\beta P_R \Lambda^3 A^* z_w^*)$$

$$= \frac{A^{*N_T} L_T^{*\,N_R}}{N! N_R! Z_T} \frac{\Lambda}{\Lambda_w} \left\{ \frac{\left[z_w^* + 2d^*(e^{\beta\varepsilon}-1)\right]^{N+1} exp\left(-\beta P_R \Lambda^3 A^* z_w^*\right)\Big|_{2d^*}^{L_T^*}}{-\beta P_R \Lambda^3 A^*} \right.$$

$$\left. + \frac{N+1}{\beta P_R \Lambda^3 A^*} \int_{2d^*}^{L_T^*} dz_w^* \left[z_w^* + 2d^*\left(e^{\beta\varepsilon} - 1\right)\right]^N exp(-\beta P_R \Lambda^3 A^* z_w^*) \right\}$$

$$= \frac{N+1}{\beta P_R \Lambda^3 A^*}. \qquad (1.17)$$

When the following limits are taken, i.e., $N \to \infty$ and $A^* \to \infty$ with N/A^* remaining finite, the first term after integration by part vanishes and we obtain the final result in Eq (1.17), which gives immediately,

$$\langle z_w^* \rangle = \frac{N}{\beta P_R \Lambda^3 A^*} - 2d^*\left(e^{\beta\varepsilon} - 1\right) = \bar{z}_w^*. \qquad \text{(compare this equation with Eq. (1.10))} \qquad (1.18)$$

In all the above calculations for the partition function and the average pore width, we assumed that $\langle z_w^* \rangle > 2d^*$, this sets the following condition on the pressure,

$$\frac{\beta P_R d A}{N} < \frac{1}{2e^{\beta\varepsilon}}. \qquad (1.19)$$

The integral adsorption strain, $\widehat{\Delta}$, is defined as the variation of the thickness of the slip pore with the confined fluid with respect to that of the bulk counterpart with the same quantity of fluid (i.e., same fluid number) and same surface area. From Eq. (1.18), we obtain immediately,

$$\widehat{\Delta} = \frac{1}{2}\left[\langle z_w \rangle - \frac{N}{\beta P_R A}\right] = -d\left(e^{\beta\varepsilon} - 1\right) \;. \qquad (1.20)$$

Eq. (1.20) shows that $\widehat{\Delta}$ depends neither on A nor on N, so we have $\widehat{\Delta} = \Delta$ in this case.

1.1.3. Differential and integral chemical potentials ($\langle z_w \rangle > 2d$)

From Eq. (1.16), we obtain immediately the following result for differential chemical potential,

$$\beta\mu = \left[\frac{\partial(\beta G)}{\partial N}\right]_{T,P_R,\mathcal{A}} = ln(\beta P_R \Lambda^3), \qquad (1.21)$$

which is equal to that of the bulk fluid in the reservoir, $\mu = \mu^{bulk}$. Thus, the disjoining chemical potential is zero in this case, $\varpi = 0$, corresponding to the case of vanishing disjoining pressure, $\Pi = 0$, when the grand potential is considered. The integral chemical potential is given by,

$$\beta\hat{\mu} = \frac{\beta G}{N} = ln(\beta P_R \Lambda^3) - \frac{2d\beta P_R A\left(e^{\beta\varepsilon} - 1\right)}{N} \qquad (1.22)$$

In the case that N scale as A, the contribution of the second term on the RHS of Eq. (1.22) is nonnegligible and $\hat{\mu} \neq \mu$. The results of Eq. (1.21) and Eq. (1.22) show that the relation between differential and integral chemical potentials given by Eq. 10 in the main text holds perfectly.

1.1.4. Differential and integral surface tensions ($\langle z_w \rangle > 2d$)

Eq. (1.16) allows for obtaining immediately the following result for differential surface tension,

$$\beta\gamma = \left[\frac{\partial(\beta G)}{\partial \mathcal{A}}\right]_{T,P_R,N} = -d\beta P_R\left(e^{\beta\varepsilon} - 1\right) \ . \tag{1.23}$$

If we decompose the Gibbs free energy into a bulk contribution and a surface contribution as follows,

$$G(T,P,N,\mathcal{A}) = \mu^{bulk}(T,P)N + \hat{\gamma}_G\mathcal{A}, \tag{1.24}$$

where $\mu^{bulk}(T,P)$ is the chemical potential of the corresponding bulk fluid at the same *T* and p, $\hat{\gamma}_G$ is defined as the integral surface tension and the index *G* denoting it is defined from Gibbs free energy instead of grand potential. Comparing Eq. (1.24) and Eq. (116), we obtain immediately,

$$\beta\hat{\gamma}_G = -d\beta P_R\left(e^{\beta\varepsilon} - 1\right). \tag{1.25}$$

Eq. (1.23) and Eq. (1.25) show that differential and integral surface tensions are identical for the weakly and normal confined situations.

1.1.5. Fluid density profile, pressure tensor, surface tension from mechanical definition ($\langle z_w \rangle > 2d$)

The fluid-wall interaction induces the inhomogeneity in the direction perpendicular to pore walls and the fluid density profile is given by,

$$\rho(z) = \left\langle \sum_{i=1}^{N} \delta(x_i - x)\delta(y_i - y)\delta(z_i - z) \right\rangle$$

$$= \frac{1}{Z_T \Lambda^{3N} \Lambda^{3N_R} \Lambda_w N! N_R!} \int_{2d}^{L_T} dz_w \int_V d\boldsymbol{r}^N \sum_{i=1}^{N} \delta(x_i - x)\delta(y_i - y)\delta(z_i - z)\, e^{-\beta \sum_{i=1}^{N} V_{fw}(z_i)} \int_{V_R} d\boldsymbol{r}^{N_R}$$

$$= \frac{N e^{-\beta V_{fw}(z)}}{Z_T \Lambda^{3N} \Lambda^{3N_R} \Lambda_w N! N_R!} \int_{2d}^{L_T} dz_w \int_V d\boldsymbol{r}^{N-1} e^{-\beta \sum_{i=1}^{N-1} V_{fw}(z_i)} \int_{V_R} d\boldsymbol{r}^{N_R}$$

$$= \frac{Z_T(N-1)}{Z_T(N)\Lambda^3} e^{-\beta V_{fw}(z)} = \beta P_R e^{-\beta V_{fw}(z)}, \tag{1.26}$$

where $\delta(x_i - x)$, $\delta(y_i - y)$, $\delta(z_i - z)$ are Dirac δ-functions, *V* and V_R are respectively the volumes of the pore and the reservoir, $Z_T(N)$ and $Z_T(N-1)$ are respectively the total partition function of the composed system with *N* and *N-1* fluid particles in the slit pore.

Due to the inhomogeneity near the walls, pressure tensor has different transversal and normal components. Since there is no fluid-fluid interaction nor fluid-wall forces in the directions parallel to the walls, the transversal component contains only a kinetic contribution and is given by,

$$\beta p_T(z) = \rho(z). \tag{1.27}$$

For the normal pressure, we have to deal with forces due to fluid-wall interactions. The normal

pressure exerting on a plan located outside the range of fluid-wall interaction, i.e., $d < z < \langle z_w \rangle - d$, has only a kinetic contribution, i.e.,

$$\beta p_N(z) = \rho(z) = \beta P_R \qquad d < z < \langle z_w \rangle - d. \tag{1.28}$$

In a region within the range of fluid-wall interactions, we have to take the fluid-wall interaction forces into account[3] and a particular attention must be paid to treat properly the discontinuity in the square-well potential. In the region, $0 < z < d$, we have,

$$\begin{aligned}
\beta p_N(z) &= \rho(z) + \frac{\beta}{A}\left\langle \sum_{i=1}^{N} F_L(z_i)\theta(z_i - z)\right\rangle. \\
&= \rho(z) + \frac{\beta}{AZ_T \Lambda^{3N} \Lambda^{3N_R} \Lambda_w N! N_R!} \int_{2d}^{L_T} dz_w \int_V d\boldsymbol{r}^N \sum_{i=1}^{N} F_L(z_i)\theta(z_i - z)\, e^{-\beta \sum_{i=1}^{N} V_{fw}(z_i)} \int_{V_R} d\boldsymbol{r}^{N_R} \\
&= \rho(z) + \beta \int_z^{\langle z_w \rangle} dz_1 \rho(z_1) F_L(z_1) \\
&= \rho(z) + \int_z^{\langle z_w \rangle} dz_1 e^{\beta\phi(z_1)} \rho(z_1) \frac{de^{-\beta\phi(z_1)}}{dz_1} \\
&= \rho(z) + \beta P_R \int_z^{\langle z_w \rangle} dz_1 \left(1 - e^{\beta\varepsilon}\right)\delta(z_1 - d) \\
&= \beta P_R e^{\beta\varepsilon} + \beta P_R\left(1 - e^{\beta\varepsilon}\right) = \beta P_R.
\end{aligned} \tag{1.29}$$

where $F_L(z_i) = -\phi'(z_i)$ is the force exerted on the *i*th fluid particle by the left wall located at $z = 0$. In a similar way, we find the following result for $p_N(z)$ in the region, $\langle z_w \rangle - d < z < \langle z_w \rangle$,

$$\begin{aligned}
\beta p_N(z) &= \rho(z) - \frac{\beta}{A}\left\langle \sum_{i=1}^{N} F_R(z_i)\theta(z - z_i)\right\rangle. \\
&= \rho(z) - \beta \int_0^{z} dz_1 \rho(z_1) F_R(z_1) \\
&= \rho(z) - \int_0^{z} dz_1 e^{\beta\phi(\langle z_w \rangle - z_1)} \rho(z_1) \frac{de^{-\beta\phi(\langle z_w \rangle - z_1)}}{dz_1} \\
&= \rho(z) - \beta P_R \int_0^{z} dz_1 \left(e^{\beta\varepsilon} - 1\right)\delta(z_1 - \langle z_w \rangle + d) \\
&= \beta P_R e^{\beta\varepsilon} - \beta P_R\left(e^{\beta\varepsilon} - 1\right) = \beta P_R.
\end{aligned} \tag{1.30}$$

where $F_R(z_i)$ is the force exerted on the *i*th fluid particle by the right wall located at $z = \langle z_w \rangle$. Eq. (1.28) – Eq. (1.30) show that the normal pressure, $p_N(z)$, is constant everywhere inside the slit pore and equal to that in the reservoir as required with a *pTN*-ensemble.

From the mechanical definition, we obtain the following result for the surface tension,

$$\begin{aligned}
\beta\gamma^{Mech} &= \frac{\beta}{2}\int_0^{\langle z_w \rangle} dz[p_N(z) - p_T(z)] \\
&= \frac{\beta P_R}{2}\int_0^{\langle z_w \rangle} dz\left[1 - e^{-\beta V_{fw}(z)}\right] = -d\beta P_R\left(e^{\beta\varepsilon} - 1\right).
\end{aligned} \tag{1.31}$$

where the factor $1/2$ accounts for the two fluid-wall interfaces with a total area of $2A$. Eq. (1.31), Eq. (1.23) and Eq. (1.25) show that γ^{Mech}, γ and $\hat{\gamma}_G$ are all equal for weakly and normally confined fluids.

Supplementary note 1.2. Strongly confined situations ($d < \langle \mathrm{z_w} \rangle < 2\mathrm{d}$)

In this subsection, we consider the situation that the piston (right wall) is located in the region, $d < \mathrm{z_w} < 2\mathrm{d}$. Thus, the fluid particles in the middle region, $\mathrm{z_w} - d < z < \mathrm{d}$, interact simultaneously with both the walls.

1.2.1. Total partition function and Gibbs free energy of the confined fluid ($d < \langle \mathrm{z_w} \rangle < 2\mathrm{d}$)

As for the previous case, we start by determining the total partition function of the composed system,

$$Z_T = \frac{A^{*N_T} e^{\beta\varepsilon N}}{N!N_R!}\frac{\Lambda}{\Lambda_w}\int_{d^*}^{2d^*} dz_w^*\left[\left(2-e^{\beta\varepsilon}\right)z_w^* + 2d^*\left(e^{\beta\varepsilon}-1\right)\right]^N \left(L_T^* - z_w^*\right)^{N_R}$$

$$= \frac{A^{*N_T} L_T^{*N_R} e^{\beta\varepsilon N}}{N!N_R!}\frac{\Lambda}{\Lambda_w}\int_{2d^*}^{2d^*} dz_w^*\left[\left(2-e^{\beta\varepsilon}\right)z_w^* + 2d^*\left(e^{\beta\varepsilon}-1\right)\right]^N \left(1 - \frac{z_w^*}{L_T^*}\right)^{N_R}$$

$$= \frac{A^{*N_T} L_T^{*N_R} e^{\beta\varepsilon N}}{N!N_R!}\frac{\Lambda}{\Lambda_w}\int_{d^*}^{2d^*} dz_w^*\left[\left(2-e^{\beta\varepsilon}\right)z_w^* + 2d^*\left(e^{\beta\varepsilon}-1\right)\right]^N exp\left(-\beta P_R \Lambda^3 A^* z_w^*\right)$$

$$= \frac{A^{*N_T} L_T^{*N_R} e^{\beta\varepsilon N}}{N!N_R!}\frac{\Lambda}{\Lambda_w}\int_{d^*}^{2d^*} dz_w^* exp\left[-\mathcal{H}(z_w^*)\right], \quad (1.32)$$

where

$$\mathcal{H}(z_w^*) = \beta P_R \Lambda^3 A^* z_w^* - N ln\left[\left(2-e^{\beta\varepsilon}\right)z_w^* + 2d^*\left(e^{\beta\varepsilon}-1\right)\right] . \quad (1.33)$$

The integral in Eq. (1.32) is again calculated with the help of Laplace method.

$$\mathcal{H}'(z_w^*) = \beta P_R \Lambda^3 A^* - \frac{N\left(2-e^{\beta\varepsilon}\right)}{\left(2-e^{\beta\varepsilon}\right)z_w^* + 2d^*\left(e^{\beta\varepsilon}-1\right)} , \quad (1.34)$$

$$\mathcal{H}''(z_w^*) = \frac{N\left(2-e^{\beta\varepsilon}\right)^2}{\left[\left(2-e^{\beta\varepsilon}\right)z_w^* + 2d^*\left(e^{\beta\varepsilon}-1\right)\right]^2} , \quad (1.35)$$

$$\bar{z}_w^* = \frac{N}{\beta P_R \Lambda^3 A^*} - \frac{2d^*\left(e^{\beta\varepsilon}-1\right)}{2-e^{\beta\varepsilon}} , \quad (1.36)$$

$$\mathcal{H}(\bar{z}_w^*) = N - \frac{2d^*\beta P_R \Lambda^3 A^*\left(e^{\beta\varepsilon}-1\right)}{2-e^{\beta\varepsilon}} - N ln\frac{N\left(2-e^{\beta\varepsilon}\right)}{\beta P_R \Lambda^3 A^*} , \quad (1.37)$$

$$Z_T = \frac{A^{*N_T} L_T^{*N_R}}{N!N_R!}\frac{\Lambda}{\Lambda_w} exp\left[\beta\varepsilon N - \mathcal{H}(\bar{z}_w^*)\right]\sqrt{\frac{2\pi}{\mathcal{H}''(\bar{z}_w^*)}}$$

$$= \frac{A^{*N_T} L_R^{*N_R}\left(1 + \frac{\langle z_w^* \rangle}{L_R^*}\right)^{N_R}}{N!N_R!}\frac{\Lambda}{\Lambda_w} exp\left[\beta\varepsilon N - \mathcal{H}(\bar{z}_w^*)\right]\sqrt{\frac{2\pi}{\mathcal{H}''(\bar{z}_w^*)}}$$

$$= \frac{A^{*N_T} L_R^{*N_R}\left(1 + \frac{N_R A^* \langle z_w^* \rangle}{A^* L_R^* N_R}\right)^{N_R}}{N!N_R!}\frac{\Lambda}{\Lambda_w} exp\left[\beta\varepsilon N - \mathcal{H}(\bar{z}_w^*)\right]\sqrt{\frac{2\pi}{\mathcal{H}''(\bar{z}_w^*)}}$$

$$= Z_R \frac{A^{*N}}{N!} exp[\beta\varepsilon N + \beta P_R A\langle z_w\rangle - \mathcal{H}(\bar{z}_w^*)] \frac{\Lambda}{\Lambda_w}\sqrt{\frac{2\pi}{\mathcal{H}''(\bar{z}_w^*)}} \ , \quad (1.38)$$

where

$$Z_R = \frac{A^{N_R} L_R^{N_R}}{\Lambda^{3N_R} N_R!}. \quad (1.39)$$

Now, the Gibbs free energy of the confined fluid can be readily obtained,

$$\beta G(T,P,N,\mathcal{A}) = \beta(F_T - F_R + P_R A\langle z_w\rangle) = -\ln\frac{Z_T}{Z_R} + \beta P_R A\langle z_w\rangle$$

$$= \ln N! - N\ln A^* - \beta\varepsilon N + \mathcal{H}(\bar{z}_w^*)$$

$$= N\ln\frac{\beta P_R \Lambda^3}{2-e^{\beta\varepsilon}} - \beta\varepsilon N - \frac{2d\beta P_R A(e^{\beta\varepsilon}-1)}{2-e^{\beta\varepsilon}} \ . \quad (1.40)$$

It is important to note that due to the presence of the term, $\ln(2-e^{\beta\varepsilon})$, Eq. (1.40) gives physically meaningful results only under the following condition when $\varepsilon > 0$ (attractive fluid-wall interactions),

$$\frac{k_B T}{\varepsilon} > \frac{1}{ln2} \ . \quad (1.41)$$

In the present study, we will consider always temperatures satisfying this inequality when $\varepsilon > 0$.

1.2.2. Average pore width and adsorption strain ($d < \langle z_w\rangle < 2d$)

To obtain the average pore width, $\langle z_w^*\rangle$, we calculate first $\langle(2-e^{\beta\varepsilon})z_w^* + 2d^*(e^{\beta\varepsilon}-1)\rangle$,

$$\langle(2-e^{\beta\varepsilon})z_w^* + 2d^*(e^{\beta\varepsilon}-1)\rangle = \frac{A^{*N_T}e^{\beta\varepsilon N}}{N!N_R!Z_T}\frac{\Lambda}{\Lambda_w}\int_{d^*}^{2d^*} dz_w^*\left[(2-e^{\beta\varepsilon})z_w^* + 2d^*(e^{\beta\varepsilon}-1)\right]^{N+1} \times$$

$$(L_T^* - z_w^*)^{N_R}$$

$$= \frac{A^{*N_T}L_T^{*N_R}e^{\beta\varepsilon N}}{N!N_R!Z_T}\frac{\Lambda}{\Lambda_w}\int_{d^*}^{2d^*} dz_w^*\left[(2-e^{\beta\varepsilon})z_w^* + 2d^*(e^{\beta\varepsilon}-1)\right]^{N+1} exp(-\beta P_R\Lambda^3 A^* z_w^*)$$

$$= \frac{A^{*N_T}L_T^{*N_R}e^{\beta\varepsilon N}}{N!N_R!Z_T}\frac{\Lambda}{\Lambda_w}\left\{\frac{\left[(2-e^{\beta\varepsilon})z_w^*+2d^*(e^{\beta\varepsilon}-1)\right]^{N+1} exp(-\beta P_R\Lambda^3 A^* z_w^*)\Big|_{d^*}^{2d^*}}{-\beta P_R\Lambda^3 A^*}\right.$$

$$\left. + \frac{(N+1)(2-e^{\beta\varepsilon})}{\beta P_R\Lambda^3 A^*}\int_{d^*}^{2d^*} dz_w^*\left[(2-e^{\beta\varepsilon})z_w^* + 2d^*(e^{\beta\varepsilon}-1)\right]^{N} exp(-\beta P_R\Lambda^3 A^* z_w^*)\right\}$$

$$= \frac{(N+1)(2-e^{\beta\varepsilon})}{\beta P_R\Lambda^3 A^*}. \quad (1.42)$$

We obtain the final result in Eq. (1.42) if the first term of the integration by part vanishes, Now, we examine in more details the conditions under which this term indeed vanishes.

$$\frac{\left[(2-e^{\beta\varepsilon})z_w^*+2d^*(e^{\beta\varepsilon}-1)\right]^{N+1} exp(-\beta P_R\Lambda^3 A^* z_w^*)\Big|_{d^*}^{2d^*}}{-\beta P_R\Lambda^3 A^*} = \frac{(2d^*)^{N+1}exp(-2\beta P_R\Lambda^3 A^* d^*)}{-\beta P_R\Lambda^3 A^*}$$

$$+\frac{{d^*}^{N+1} exp[-\beta P_R \Lambda^3 A^* d^* + (N+1)\beta\varepsilon]}{\beta P_R \Lambda^3 A^*}. \tag{1.43}$$

The first term on the RHS of Eq. (1.43) vanishes in the limits, $N \to \infty$ and $A^* \to \infty$, with $n^* = N/A^*$ remaining finite. If $\varepsilon < 0$ (repulsive fluid-wall interaction), the second term on the RHS of Eq. (1.43) vanishes also in the thermodynamic limits. But, for $\varepsilon > 0$ (attractive fluid-wall interaction), the second term on the RHS of Eq. (1.43) vanishes in the thermodynamic limits under the following additional condition,

$$\frac{\beta P_R A d}{N+1} > \beta\varepsilon, \qquad \varepsilon > 0. \tag{1.44}$$

We will need this condition in some later discussions.

From Eq. (1.42), we obtain readily,

$$\langle z_w^* \rangle = \frac{N+1}{\beta P_R \Lambda^3 A^*} - \frac{2d^*(e^{\beta\varepsilon}-1)}{2-e^{\beta\varepsilon}} = \bar{z}_w^*. \qquad \text{[compare with Eq. (1.36)]} \tag{1.45}$$

It is recalled that we consider here the pore width in the region of $d^* < \langle z_w^* \rangle < 2d^*$, so we have,

$$1 < \frac{N+1}{\beta P_R A d} - \frac{2(e^{\beta\varepsilon}-1)}{2-e^{\beta\varepsilon}} < 2, \tag{1.46}$$

which can be rewritten as,

$$\frac{2-e^{\beta\varepsilon}}{2} < \frac{\beta P_R A d}{N+1} < \frac{2-e^{\beta\varepsilon}}{e^{\beta\varepsilon}}. \tag{1.47}$$

For attractive fluid-wall interactions ($\varepsilon > 0$), the inequality (1.44) must be satisfied also. The inequalities (1.44) and (1.47) can hold simultaneously under the following condition,

$$\beta\varepsilon < \frac{2-e^{\beta\varepsilon}}{2}, \qquad \Rightarrow \qquad e^{\beta\varepsilon} + 2\beta\varepsilon < 2 \ . \tag{1.48}$$

The above inequality holds for $e^{\beta\varepsilon} < 1.37015$. Finally, we find the following conditions for the pore width to be in the region of $d^* < \langle z_w^* \rangle < 2d^*$,

$$\frac{2-e^{\beta\varepsilon}}{2} < \frac{\beta P_R A d}{N+1} < \frac{2-e^{\beta\varepsilon}}{e^{\beta\varepsilon}} \ , \qquad T > \frac{1}{ln 1.37015} \frac{\varepsilon}{k_B} = 3.1754 \frac{\varepsilon}{k_B} \ . \tag{1.49}$$

From Eq. (1.45), we obtain readily the following result for integral adsorption strain,

$$\hat{\Delta} = \frac{1}{2}\left[\langle z_w \rangle - \frac{N}{\beta P_R A}\right] = -\frac{d(e^{\beta\varepsilon}-1)}{2-e^{\beta\varepsilon}} \ . \tag{1.50}$$

Eq. (1.50) shows that $\hat{\Delta}$ depends neither on $\mathcal{A}$ nor on N, so we have again $\hat{\Delta} = \Delta$ for the strong confined situation.

1.2.3. Differential, disjoining and integral chemical potentials ($d < \langle z_w \rangle < 2d$)

From Eq. (1.40), we obtain readily the following result for differential chemical potential,

$$\beta\mu = \left[\frac{\partial(\beta G)}{\partial N}\right]_{T,P_R,\mathcal{A}} = ln(\beta P_R \Lambda^3) - \beta\varepsilon - ln\left(2 - e^{\beta\varepsilon}\right) = ln(\beta P_R \Lambda^3) + \beta\varpi \ , \tag{1.51}$$

which is not equal to the chemical potential of the corresponding bulk fluid. We define the difference as disjoining chemical potential, i.e.,

$$\varpi = -\varepsilon - k_B T ln\left(2 - e^{\beta\varepsilon}\right) \ . \tag{1.52}$$

It is to note that disjoining chemical potential is only a function of temperature but does not depend on pressure. Eq. (1.51) shows that the differential chemical potential is only function of T and P_R but does not depend on $N/\mathcal{A}$. From Eq. (1.40), we obtain readily the integral chemical potential,

$$\beta\hat{\mu} = \frac{\beta G}{N} = ln\frac{\beta P_R \Lambda^3}{2 - e^{\beta\varepsilon}} - \beta\varepsilon - \frac{2d\beta P_R A\left(e^{\beta\varepsilon} - 1\right)}{\left(2 - e^{\beta\varepsilon}\right)N} = \beta\mu - \frac{2d\beta P_R A\left(e^{\beta\varepsilon} - 1\right)}{\left(2 - e^{\beta\varepsilon}\right)N}$$

$$= \beta\mu^{bulk} + \beta\varpi - \frac{2d\beta P_R A\left(e^{\beta\varepsilon} - 1\right)}{\left(2 - e^{\beta\varepsilon}\right)N}. \tag{1.53}$$

The above results show that for strongly confined fluids, the three chemical potentials are all different from each other, i.e., $\mu \neq \mu^{bulk} \neq \hat{\mu}$.

1.2.4. Differential and integral surface tensions ($d < \langle z_w \rangle < 2d$)

From Eq. (1.40), we obtain immediately the following result for the differential surface tension,

$$\beta\gamma = \left[\frac{\partial(\beta G)}{\partial \mathcal{A}}\right]_{T,P_R,N} = -\frac{d\beta P_R\left(e^{\beta\varepsilon} - 1\right)}{2 - e^{\beta\varepsilon}} \ . \tag{1.54}$$

The integral surface tension is given by,

$$\beta\hat{\gamma}_G = \frac{\beta\left(G - \mu^{bulk} N\right)}{\mathcal{A}} = -\frac{d\beta P_R\left(e^{\beta\varepsilon} - 1\right)}{2 - e^{\beta\varepsilon}} - \frac{N}{\mathcal{A}}\left[\beta\varepsilon + ln\left(2 - e^{\beta\varepsilon}\right)\right] = \beta\gamma + \frac{\beta\varpi N}{\mathcal{A}} \ . \tag{1.55}$$

This relation shows that the disjoining chemical potential allows for relating the differential surface tension to the integral surface tension, thus confirms Eq. 12 in the main text derived from thermodynamics. Eq. (1.55) shows clearly that the integral surface tension depends not only on T and P but also on $N/\mathcal{A}$. In our recent work[1], we have obtained the following result for integral surface tension from grand potential,

$$\beta\hat{\gamma}_\Omega = -\frac{V}{\mathcal{A}}\frac{e^{\beta\mu}}{\Lambda^3}\left(e^{\beta\varepsilon} - 1\right)\left[1 + \left(\frac{d\mathcal{A}}{V} - 1\right)e^{\beta\varepsilon}\right] \ . \tag{1.56}$$

Now, we will show that $\hat{\gamma}_G$, is different from $\hat{\gamma}_\Omega$. For this, it is necessary to rewrite Eq. (1.55) in terms of μ and V instead of N and P_R. First, we can express the pressure in terms of chemical potential with the help of Eq. (1.51), i.e.,

$$\beta P_R = \frac{e^{\beta(\mu + \varepsilon)}}{\Lambda^3}\left(2 - e^{\beta\varepsilon}\right) \ . \tag{1.57}$$

Then, using Eq. (1.45) and Eq. (1.57), we can express $N/\mathcal{A}$ in terms of volume, V and μ, i.e.,

$$\frac{N}{\mathcal{A}} = \beta P_R \frac{V}{\mathcal{A}} + \frac{d\beta P_R\left(e^{\beta\varepsilon}-1\right)}{2-e^{\beta\varepsilon}} = \frac{e^{\beta(\mu+\varepsilon)}}{\Lambda^3}\left[\frac{V}{\mathcal{A}}\left(2-e^{\beta\varepsilon}\right)+d\left(e^{\beta\varepsilon}-1\right)\right] \ . \tag{1.58}$$

Eq. (1.57) is used when going to the second equality of Eq. (1.58). Substituting Eq. (1.57) and Eq. (1.58) into Eq. (1.55), we obtain,

$$\beta\hat{\gamma}_G = -\frac{e^{\beta(\mu+\epsilon)}}{\Lambda^3}\left[d\left(e^{\beta\varepsilon}-1\right)(1-\beta\varpi)-\beta\varpi\left(2-e^{\beta\varepsilon}\right)\frac{V}{\mathcal{A}}\right]$$

$$= -\frac{V}{\mathcal{A}}\frac{e^{\beta(\mu+\epsilon)}}{\Lambda^3}\left(e^{\beta\varepsilon}-1\right)\left[\frac{d\mathcal{A}}{V}(1-\beta\varpi)-\beta\varpi\frac{\left(2-e^{\beta\varepsilon}\right)}{e^{\beta\varepsilon}-1}\right] \ . \tag{1.59}$$

Comparing Eq. (1.59) with Eq. (1.56), we see that $\hat{\gamma}_G \neq \hat{\gamma}_\Omega$, this confirms the ensemble-dependence of integral surface tension. Unlike differential surface tension, the integral surface tension calculated for a specific ensemble cannot be used for another ensemble. The formal relation between $\hat{\gamma}_G$ and $\hat{\gamma}_\Omega$ given by Eq. 41 in the main text holds perfectly, i.e.,

$$\beta\hat{\gamma}_\Omega = \beta\hat{\gamma}_G - \frac{N\beta\varpi+\beta\Pi V}{\mathcal{A}} = \beta\gamma - \frac{\beta\Pi V}{\mathcal{A}} = -\frac{d\beta P_R\left(e^{\beta\varepsilon}-1\right)}{2-e^{\beta\varepsilon}} + \frac{V}{\mathcal{A}}\left(e^{\beta\varepsilon}-1\right)^2\frac{e^{\beta\mu}}{\Lambda^3}$$

$$= -\frac{e^{\beta(\mu+\epsilon)}}{\Lambda^3}d\left(e^{\beta\varepsilon}-1\right)+\frac{V}{\mathcal{A}}\left(e^{\beta\varepsilon}-1\right)^2\frac{e^{\beta\mu}}{\Lambda^3}$$

$$= -\frac{V}{\mathcal{A}}\frac{e^{\beta\mu}}{\Lambda^3}\left(e^{\beta\varepsilon}-1\right)\left[\frac{d\mathcal{A}}{V}e^{\beta\varepsilon}-e^{\beta\varepsilon}+1\right] \ . \tag{1.60}$$

To obtain the final result in Eq. (1.60), we used Eq. (1.57) to express P_R in terms of chemical potential and the following result for the disjoining pressure obtained in Ref. (1),

$$\beta\Pi = -\left(e^{\beta\varepsilon}-1\right)^2\frac{e^{\beta\mu}}{\Lambda^3} \ . \tag{1.61}$$

The final result in Eq. (1.60) is indeed identical to Eq. (1.56).

Taking derivative on the both sides of Eq. (1.54) with respect to pressure, we obtain,

$$\left[\frac{\partial(\beta\gamma)}{\partial(\beta P_R)}\right]_T = -\frac{d\left(e^{\beta\varepsilon}-1\right)}{2-e^{\beta\varepsilon}} = \Delta \ . \tag{1.62}$$

From Eq. (1.55), we obtain,

$$\left[\frac{\partial(\beta\hat{\gamma}_G)}{\partial(\beta P_R)}\right]_{T,N/\mathcal{A}} = \left[\frac{\partial(\beta\gamma)}{\partial(\beta P_R)}\right]_T = \Delta \ , \tag{1.63}$$

(note that $\hat{\Delta} = \Delta$ for the model considered here). The validity of the new generalized adsorption equations (Eq. 18 and Eq. 21 in the main text) are perfectly illustrated by Eq. (1.62) and Eq. (1.63).

1.2.5. Fluid density profile, pressure tensor, surface tension from mechanical definition ($d < \langle z_w \rangle < 2d$)

The fluid density profile is given by,

$$\rho(z) = \frac{Z_T(N-1)}{Z_T(N)\Lambda^3} e^{-\beta V_{fw}(z)} = \frac{\beta P_R}{e^{\beta\varepsilon}\left(2-e^{\beta\varepsilon}\right)} e^{-\beta V_{fw}(z)} \quad . \tag{1.64}$$

Although the expression after the first equality is formally the same as that in Eq. (1.26), $Z_T(N-1)/Z_T(N)$ gives a different result for the strongly confined situation considered here (comparing the final result in Eq. (1.64) with that of Eq. (1.26)).

Since there is no fore in the direction parallel to wall surfaces, the transversal component of the pressure tensor has only the kinetic contribution, i.e.,

$$\beta p_T(z) = \rho(z). \tag{1.65}$$

Due to fluid-wall interactions, we need to calculate again the normal component of the pressure tensor in three different regions. In the region $0 < z < \langle z_w \rangle - d$, only the interaction between the fluid and the left wall contributes, the normal component of the pressure tensor is given by,

$$\beta p_N(z) = \rho(z) + \frac{\beta}{A}\left\langle \sum_{i=1}^N F_L(z_i)\theta(z_i - z)\right\rangle.$$

$$= \rho(z) + \frac{\beta}{A Z_T \Lambda^{3N}\Lambda^{3N_R}\Lambda_w N! N_R!}\int_d^{2d} dz_w \int_V d\boldsymbol{r}^N \sum_{i=1}^N F_L(z_i)\theta(z_i - z)\, e^{-\beta\sum_{i=1}^N V_{fw}(z_i)} \int_{V_R} d\boldsymbol{r}^{N_R}$$

$$= \rho(z) + \beta \int_z^{\langle z_w \rangle} dz_1 \rho(z_1) F_L(z_1)$$

$$= \rho(z) + \int_z^{\langle z_w \rangle} dz_1 e^{\beta\phi(z_1)} \rho(z_1) \frac{de^{-\beta\phi(z_1)}}{dz_1}$$

$$= \rho(z) + \frac{\beta P_R}{e^{\beta\varepsilon}\left(2-e^{\beta\varepsilon}\right)} \int_z^{\langle z_w \rangle} dz_1 e^{\beta\phi(\langle z_w \rangle - z_1)}\left(1-e^{\beta\varepsilon}\right)\delta(z_1 - d)$$

$$= \frac{\beta P_R}{2-e^{\beta\varepsilon}} + \frac{\beta P_R}{2-e^{\beta\varepsilon}}\left(1-e^{\beta\varepsilon}\right) = \beta P_R. \tag{1.66}$$

In the region $d < z < \langle z_w \rangle$, only the interaction between the fluid and the right wall contributes, we obtain the following result for $p_N(z)$,

$$\beta p_N(z) = \rho(z) - \frac{\beta}{A}\left\langle \sum_{i=1}^N F_R(z_i)\theta(z - z_i)\right\rangle.$$

$$= \rho(z) - \beta \int_0^z dz_1 \rho(z_1) F_R(z_1)$$

$$= \rho(z) - \int_0^z dz_1 e^{\beta\phi(\langle z_w \rangle - z_1)} \rho(z_1) \frac{de^{-\beta\phi(\langle z_w \rangle - z_1)}}{dz_1}$$

$$= \rho(z) - \frac{\beta P_R}{e^{\beta\varepsilon}\left(2-e^{\beta\varepsilon}\right)} \int_0^z dz_1 e^{\beta\phi(z_1)} \frac{de^{-\beta\phi(\langle z_w \rangle - z_1)}}{dz_1}$$

$$= \rho(z) - \frac{\beta P_R}{2-e^{\beta\varepsilon}} \int_0^z dz_1 \left(e^{\beta\varepsilon}-1\right)\delta(z_1 - \langle z_w \rangle + d)$$

$$= \frac{\beta P_R}{2-e^{\beta\varepsilon}} - \frac{\beta P_R}{2-e^{\beta\varepsilon}}\left(e^{\beta\varepsilon}-1\right) = \beta P_R. \tag{1.67}$$

In the middle region $\langle z_w \rangle - d < z < d$, we have to take into account the interaction of the fluid with both walls for $p_N(z)$, i.e.,

$$\beta p_N(z) = \rho(z) + \frac{\beta}{A}\left\langle \sum_{i=1}^{N} F_L(z_i)\theta(z_i - z)\right\rangle - \frac{\beta}{A}\left\langle \sum_{i=1}^{N} F_R(z_i)\theta(z - z_i)\right\rangle.$$

$$= \rho(z) + \beta \int_z^{z_w} dz_1 \rho(z_1) F_L(z_1) - \beta \int_0^z dz_1 \rho(z_1) F_R(z_1)$$

$$= \rho(z) + \int_z^{\langle z_w \rangle} dz_1 e^{\beta\phi(z_1)} \rho(z_1) \frac{de^{-\beta\phi(z_1)}}{dz_1} - \int_0^z dz_1 e^{\beta\phi(\langle z_w \rangle - z_1)} \rho(z_1) \frac{de^{-\beta\phi(\langle z_w \rangle - z_1)}}{dz_1}$$

$$= \rho(z) + \frac{\beta P_R}{e^{\beta\varepsilon}(2-e^{\beta\varepsilon})}\left[\int_z^{\langle z_w \rangle} dz_1 e^{\beta\phi(\langle z_w \rangle - z_1)} \frac{de^{-\beta\phi(z_1)}}{dz_1} - \int_0^z dz_1 e^{\beta\phi(z_1)} \frac{de^{-\beta\phi(\langle z_w \rangle - z_1)}}{dz_1}\right]$$

$$= \rho(z) + \frac{\beta P_R}{e^{\beta\varepsilon}(2-e^{\beta\varepsilon})} \int_z^{\langle z_w \rangle} dz_1 e^{\beta\phi(\langle z_w \rangle - z_1)} \left(1 - e^{\beta\epsilon}\right) \delta(z_1 - d)$$

$$- \frac{\beta P_R}{e^{\beta\varepsilon}(2-e^{\beta\varepsilon})} \int_0^z dz_1 e^{\beta\phi(z_1)} \left(e^{\beta\varepsilon} - 1\right) \delta(z_1 - \langle z_w \rangle + d)$$

$$= \frac{\beta P_R e^{\beta\varepsilon}}{2-e^{\beta\varepsilon}} + \frac{\beta P_R}{2-e^{\beta\varepsilon}}\left(1 - e^{\beta\epsilon}\right) - \frac{\beta P_R}{2-e^{\beta\varepsilon}}\left(e^{\beta\varepsilon} - 1\right) = \beta P_R \ . \quad (1.68)$$

The results of Eq. (1.66) – Eq. (1.68) show that the normal component of the pressure tensor is constant everywhere inside the slit pore and equal to that of the reservoir as we can anticipate for a *pTN*-ensemble.

Now, we calculate also surface tension from the mechanical definition, i.e.,

$$\beta\gamma^{Mech} = \frac{\beta}{2}\int_0^{\langle z_w \rangle} dz [p_N(z) - p_T(z)]$$

$$= \frac{\beta P_R}{2}\int_0^{\langle z_w \rangle} dz \left[1 - \frac{1}{e^{\beta\varepsilon}(2-e^{\beta\varepsilon})} e^{-\beta V_{fw}(z)}\right]$$

$$= \frac{\beta P_R}{2}\left[\langle z_w \rangle - \frac{\langle z_w \rangle - d}{2-e^{\beta\varepsilon}} - \frac{(2d - \langle z_w \rangle)e^{\beta\varepsilon}}{2-e^{\beta\varepsilon}} - \frac{\langle z_w \rangle - d}{2-e^{\beta\varepsilon}}\right]$$

$$= -\frac{d\beta P_R\left(e^{\beta\varepsilon}-1\right)}{2-e^{\beta\varepsilon}} \ . \quad (1.69)$$

This is the same result as that given in Eq. (1.54), so the mechanical definition gives the differential surface tension. In grand canonical ensemble, we found also that the mechanical definition gives differential surface tension[1] and this shows its ensemble-independence.

1.2.6. Different thermodynamic potentials and Legendre transforms ($d < \langle \mathrm{z_w} \rangle < 2\mathrm{d}$)

Now, we will discuss some issues concerning the environment-dependence (or ensemble-dependence) of thermodynamics for small systems. First, we examine the question if all the thermodynamic potentials describe equally well a given small system. For thermodynamics of macroscopic systems, different thermodynamic potentials are related through various Legendre transforms. We know now

that for thermodynamics of small systems, there are two types of intensive variables, i.e., differential and integral ones. The present work shows that only differential thermodynamic variables are ensemble-independent. So, it is natural that such variables should be used for Legendre transforms but not the integral thermodynamic variables. Now, we illustrate this by two successive Legendre transforms which allow for obtaining grand potential from Gibbs free energy, i.e.,

$$\beta\Omega(T,\mu,V,\mathcal{A}) = \beta[G - P_R V - \mu N] \quad . \qquad (1.70)$$

Substituting Eq. (1.40), Eq. (1.51) and Eq. (1.58) into the RHS of Eq. (1.70), we obtain,

$$\beta\Omega(T,\mu,V,\mathcal{A}) = -N = -\beta P_R V - \frac{2\beta P_R dA\left(e^{\beta\varepsilon}-1\right)}{2-e^{\beta\varepsilon}}$$

$$= -\frac{e^{\beta(\mu+\varepsilon)}}{\Lambda^3}\left(2-e^{\beta\varepsilon}\right)V - \frac{e^{\beta(\mu+\varepsilon)}}{\Lambda^3}2dA\left(e^{\beta\varepsilon}-1\right)$$

$$= -\frac{e^{\beta(\mu+\varepsilon)}}{\Lambda^3}V\left[1+\left(\frac{2dA}{V}-1\right)\left(e^{\beta\varepsilon}-1\right)\right]. \qquad (1.71)$$

Eq. (1.58) is used when going to the second equality on the RHS of Eq. (1.70), then Eq. (1.57) is used to replace P_R in terms of chemical potential. The final result in Eq. (1.71) is precisely that obtained in ref. (1) from the grand canonical ensemble.

Supplementary note 1.3. Extremely confined situations ($0 < \langle z_w \rangle \leq d$)

1.3.1. Total partition function and Gibbs free energy of confined fluid ($0 < \langle z_w \rangle \leq d$)

With the composed system depicted in Fig. 1 of the main text, we determine first the partition function of the whole system from which Gibbs free energy of the confined fluid can be obtained. Since the composed system is in a canonical ensemble, its partition function is given by,

$$Z_T = \frac{1}{\Lambda^{3N}\Lambda^{3N_R}\Lambda_w N! N_R!}\int_0^d dz_w \left\{A\int_0^{z_w} dz\, exp\left[-\beta V_{fw}(z)\right]\right\}^N \left(A\int_{z_w}^{L_T} dz\right)^{N_R}, \qquad (1.72)$$

where Λ and Λ_W are respectively the thermal wavelengths of fluid particles and the fluctuating wall (piston), A surface area of one wall, L_T width of the total system. In order to deal with only unitless quantities, we reduce all the length related quantity by the fluid thermal wavelength and the reduced quantities are denoted with a star. Thus, Eq. (1.72) becomes,

$$Z_T = \frac{{A^*}^{N_T} e^{2\beta\varepsilon N}}{N! N_R!}\frac{\Lambda}{\Lambda_w}\int_0^{d^*} dz_w^* {z_w^*}^N (L_T^* - z_w^*)^{N_R}$$

$$= \frac{{A^*}^{N_T} {L_T^*}^{N_R} e^{2\beta\varepsilon N}}{N! N_R!}\frac{\Lambda}{\Lambda_w}\int_0^{d^*} dz_w^* {z_w^*}^N \left(1 - \frac{z_w^*}{L_T^*}\right)^{N_R}$$

$$= \frac{{A^*}^{N_T} {L_T^*}^{N_R} e^{2\beta\varepsilon N}}{N! N_R!}\frac{\Lambda}{\Lambda_w}\int_0^{d^*} dz_w^* {z_w^*}^N exp(-\beta P_R \Lambda^3 A^* z_w^*)$$

$$= \frac{A^{*N_T} {L_T^*}^{N_R} e^{2\beta\varepsilon N}}{N! N_R!} \frac{\Lambda}{\Lambda_w} \int_0^{d^*} dz_w^* exp[-\mathcal{H}(z_w^*)] \ , \tag{1.73}$$

where

$$\mathcal{H}(z_w^*) = \beta P_R \Lambda^3 A^* z_w^* - N ln z_w^* . \tag{1.74}$$

$$\mathcal{H}'(z_w^*) = \beta P_R \Lambda^3 A^* - \frac{N}{z_w^*} \ . \tag{1.75}$$

$$\mathcal{H}''(z_w^*) = \frac{N}{{z_w^*}^2} \ . \tag{1.76}$$

Eqs (1.75) and (1.76) show that $\mathcal{H}(z_w^*)$ has a minimum at $\bar{z}_w^*$,

$$\bar{z}_w^* = \frac{N}{\beta P \Lambda^3 A^*} \ , \tag{1.77}$$

$$\mathcal{H}(\bar{z}_w^*) = N - N ln \frac{N}{\beta P_R \Lambda^3 A^*} \ . \tag{1.78}$$

Expanding $\mathcal{H}(z_w^*)$ around $\bar{z}_w^*$ in a Taylor series to the second order, we obtain,

$$\mathcal{H}(z_w^*) = \mathcal{H}(\bar{z}_w^*) + \frac{1}{2}\mathcal{H}''(\bar{z}_w^*)(z_w^* - \bar{z}_w^*)^2 . \tag{1.79}$$

Substituting Eq (1.79) into Eq (1.73), we obtain,

$$Z_T = \frac{A^{*N_T} {L_T^*}^{N_R} e^{2\beta\varepsilon N}}{N! N_R!} exp[-\mathcal{H}(\bar{z}_w^*)] \frac{\Lambda}{\Lambda_w} \int_0^{d^*} dz_w^* exp\left[-\frac{1}{2}\mathcal{H}''(\bar{z}_w^*)(z_w^* - \bar{z}_w^*)^2\right] \ , \tag{1.80}$$

In the limits, $N \to \infty$ and $L_T \to \infty$, the integral on the RHS of Eq (1.80) becomes a Gaussian one and we obtain finally,

$$Z_T = \frac{A^{*N_T} {L_T^*}^{N_R} e^{2\beta\varepsilon N}}{N! N_R!} exp[-\mathcal{H}(\bar{z}_w^*)] \frac{\Lambda}{\Lambda_w} \sqrt{\frac{2\pi}{\mathcal{H}''(\bar{z}_w^*)}}$$

$$= \frac{A^{*N_T} {L_R^*}^{N_R} \left(1 + \frac{\langle z_w^* \rangle}{L_R^*}\right)^{N_R} e^{2\beta\varepsilon N}}{N! N_R!} exp[-\mathcal{H}(\bar{z}_w^*)] \frac{\Lambda}{\Lambda_w} \sqrt{\frac{2\pi}{\mathcal{H}''(\bar{z}_w^*)}}$$

$$= Z_R \frac{A^{*N}}{N!} exp[-\mathcal{H}(\bar{z}_w^*) + \beta P_R \Lambda^3 \langle z_w^* \rangle A^* + 2\beta\varepsilon N] \frac{\Lambda}{\Lambda_w} \sqrt{\frac{2\pi}{\mathcal{H}''(\bar{z}_w^*)}} \ , \tag{1.81}$$

When going to the last equality of Eq (1.81), the thermodynamic limit is taken, i.e., $N_R \to \infty$ and $(AL_R) \to \infty$ with $N_R/(AL_R) = \beta P_R$ remaining finite. We obtain the Gibbs free energy of the confined fluid by subtracting the Gibbs free energy of the reservoir from that of the total system, i.e.,

$$\beta G(T, P, N, \mathcal{A}) = \beta(G_T - G_R) = \beta(F_T - F_R + P_R \langle z_w \rangle A) = -ln\frac{Z_T}{Z_R} + \beta P_R \langle z_w \rangle A$$

$$= N ln(\beta P_R \Lambda^3) - 2\beta\varepsilon N \ . \tag{1.82}$$

The term involving $\mathcal{H}''(\bar{z}_w^*)$ becomes negligible in the thermodynamic limit, $N \to \infty$ and $A \to \infty$, thus it does not contribute to the Gibbs free energy.

1.3.2. Average pore width and adsorption strain (**$0 < \langle z_w \rangle \leq d$**)

$$\langle z_w^* \rangle = \frac{A^{*N_T} L_T^{*\,N_R} e^{2\beta\varepsilon N}}{N! N_R! Z_T} \frac{\Lambda}{\Lambda_w} \int_0^{d^*} dz_w^* z_w^{*\,N+1} exp(-\beta P_R \Lambda^3 A^* z_w^*)$$

$$= \frac{A^{*N_T} L_T^{*\,N_R} e^{2\beta\varepsilon N}}{N! N_R! Z_T} \frac{\Lambda}{\Lambda_w} \left[\frac{d^{*N+1} exp(-\beta P_R \Lambda^3 A^* d^*)}{-\beta P_R \Lambda^3 A^*} + \frac{N+1}{\beta P_R \Lambda^3 A^*} \int_0^{d^*} dz_w^* z_w^{*\,N} exp(-\beta P_R \Lambda^3 A^* z_w^*) \right]$$

$$= \frac{N+1}{\beta P_R \Lambda^3 A^*}. \quad (1.83)$$

When the following limits are taken, i.e., $N \to \infty$ and $A^* \to \infty$ with N/A^* remaining finite, the first term after integration by part vanishes and we obtain the final result in Eq (1.83), which is the same as Eq (1.77). Since Eq (1.83) is the same as the equation of state of a bulk ideal gas, the integral adsorption strain vanishes, i.e.,

$$\widehat{\Delta} = 0 \ . \quad (1.84)$$

1.3.3. Differential and integral chemical potentials (**$0 < \langle z_w \rangle \leq d$**)

From Eq. (1.82), we obtain the following result for differential and integral chemical potential,

$$\beta\mu = \left[\frac{\partial(\beta G)}{\partial N}\right]_{T,P_R,\mathcal{A}} = ln(\beta P_R \Lambda^3) - 2\beta\varepsilon = \frac{\beta G}{N} = \beta\hat{\mu}. \quad (1.85)$$

Although the differential and the integral chemical potential are the same in this case, they are not equal to that of the bulk ideal gas. So, the disjoining chemical potential is not zero and given by,

$$\varpi = \beta\mu - ln(\beta P_R \Lambda^3) = -2\beta\varepsilon \quad (1.86)$$

1.3.4. Differential and integral surface tensions (**$0 < \langle z_w \rangle \leq d$**)

Eq. (1.82) shows immediately that the differential surface tension is zero, i.e.,

$$\beta\gamma = \left[\frac{\partial(\beta G)}{\partial \mathcal{A}}\right]_{T,P_R,N} = 0 \ . \quad (1.87)$$

If we decompose the Gibbs free energy into a bulk contribution and a surface contribution as follows,

$$G(T,P,N,\mathcal{A}) = \mu^{bulk}(T,P)N + \hat{\gamma}_G \mathcal{A}, \quad (1.88)$$

where $\mu^{bulk}(T,P)$ is the chemical potential of the corresponding bulk fluid at the same *T* and p, $\hat{\gamma}_G$ is defined as the integral surface tension and the index *G* denoting it is defined from Gibbs free energy. Comparing Eq. (1.88) and Eq. (1.82), we obtain immediately,

$$\beta\hat{\gamma}_G = -2\beta\varepsilon \frac{N}{\mathcal{A}} = \frac{\varpi N}{\mathcal{A}}. \quad (1.89)$$

Although the differential surface tension is zero in this case, the integral surface tension does not vanish. It is also to note that $\hat{\gamma}_G$ does not depend on the pressure. From Eqs (1.89) and (1.84), one can check readily that the generalized Gibbs adsorption equation Eq 21 in the text holds also in this

case, i.e.,

$$\left(\frac{\partial \hat{\gamma}_G}{\partial p}\right)_{T,\hat{\varphi}} = \hat{\Delta} = 0. \tag{1.90}$$

1.3.5. Fluid density profile, pressure tensor, surface tension from mechanical definition ($0 < \langle z_w \rangle \leq d$ ***)***

In this case, the fluid-wall interaction becomes constant. So, it does not induces any inhomogeneity and the fluid density becomes constant also,

$$\begin{aligned}\rho(z) &= \left\langle \sum_{i=1}^{N} \delta(x_i - x)\delta(y_i - y)\delta(z_i - z) \right\rangle \\ &= \frac{1}{Z_T \Lambda^{3N} \Lambda^{3N_R} \Lambda_w N! N_R!} \int_0^d dz_w \int_V d\boldsymbol{r}^N \sum_{i=1}^{N} \delta(x_i - x)\delta(y_i - y)\delta(z_i - z)\, e^{-\beta \sum_{i=1}^{N} V_{fw}(z_i)} \int_{V_R} d\boldsymbol{r}^{N_R} \\ &= \frac{N e^{-\beta V_{fw}(z)}}{Z_T \Lambda^{3N} \Lambda^{3N_R} \Lambda_w N! N_R!} \int_0^d dz_w \int_V d\boldsymbol{r}^{N-1} e^{-\beta \sum_{i=1}^{N-1} V_{fw}(z_i)} \int_{V_R} d\boldsymbol{r}^{N_R} \\ &= \frac{Z_T(N-1)}{Z_T(N)\Lambda^3} e^{-\beta V_{fw}(z)} = \beta P_R e^{-\beta V_{fw}(z)} = \beta P_R e^{2\beta\varepsilon},\end{aligned} \tag{1.91}$$

where $\delta(x_i - x)$, $\delta(y_i - y)$, $\delta(z_i - z)$ are Dirac δ-functions, V and V_R are respectively the volumes of the pore and the reservoir, $Z_T(N)$ and $Z_T(N-1)$ are respectively the total partition function of the composed system with N and N-1 fluid particles in the slit pore.

Since there is no fluid-fluid interaction nor fluid-wall forces in the directions parallel to the walls, the transversal component contains only a kinetic contribution and is given by,

$$\beta p_T(z) = \rho(z). \tag{1.92}$$

In the current case, the constant fluid-wall interaction does not give any force in the direction perpendicular to the pore walls. So, the normal component of the pressure tensor is also given by the fluid density, i.e.,

$$\beta p_N(z) = \rho(z) \qquad\qquad 0 < z \leq d. \tag{1.93}$$

Thus, we obtain immediately that surface tension from the mechanical definition is zero, i.e.,

$$\beta\gamma^{Mech} = \frac{\beta}{2} \int_0^{\langle z_w \rangle} dz[p_N(z) - p_T(z)] = 0 \quad . \tag{1.94}$$

So, the mechanical definition gives again the differential surface tension in this case.

Supplementary discussion 2. Some supplementary thermodynamic results based on grand potential

In this supplementary discussion, we give some additional results about the adsorption equation for $\hat{\gamma}_\Omega$, which were not presented in ref. [1]. From Eq. 20 in the main text, we obtain readily,

$$\left[\frac{\partial \hat{\gamma}_\Omega}{\partial \hat{\ell}}\right]_{T,\mu} = -\Pi \ . \tag{2.1}$$

Then, Eq. 20 in the main text and Eq. (2.1) lead to,

$$\gamma = \hat{\gamma}_\Omega - \hat{\ell}\left[\frac{\partial \hat{\gamma}_\Omega}{\partial \hat{\ell}}\right]_{T,\mu} \ . \tag{2.2}$$

Although this relation is perfectly compatible with the following one derived in ref. [1], i.e.,

$$\gamma = \hat{\gamma}_\Omega + \mathcal{A}\left[\frac{\partial \hat{\gamma}_\Omega}{\partial \mathcal{A}}\right]_{\mu,T,V}, \tag{2.3}$$

Eq. (2.2) is a more practical one since $\hat{\gamma}_\Omega$ is a function of *T*, μ and $\hat{\ell}$ but does not depend on *V* and $\mathcal{A}$ separately. From Eq. 19 in the main text, we derive now the following adsorption equation for $\hat{\gamma}_\Omega$,

$$\left(\frac{\partial \hat{\gamma}_\Omega}{\partial \mu}\right)_{T,\hat{\ell}} = -\frac{N - N^{bulk}}{\mathcal{A}} = -\hat{\Gamma} \ , \tag{2.4}$$

which is more practical than the following one derived previously [1],

$$\left(\frac{\partial \hat{\gamma}_\Omega}{\partial \mu}\right)_{T,V,\mathcal{A}} = -\hat{\Gamma}. \tag{2.5}$$

If we define the differential adsorption as follows,

$$\Gamma = \hat{\Gamma} - \hat{\ell}\left[\frac{\partial \hat{\Gamma}}{\partial \hat{\ell}}\right]_{T,\mu} \ , \tag{2.6}$$

the μTV-ensemble adsorption equation for differential surface tension becomes,

$$\left(\frac{\partial \gamma}{\partial \mu}\right)_T = -\Gamma \ . \tag{2.7}$$

It is to be emphasized that Eq. (2.7) is identical to the original Gibbs adsorption equation only when $\gamma = \hat{\gamma}$ and $\Gamma = \hat{\Gamma}$. To close this digression, it is quite gratifying to see that the present study with *pTN*-environment is also helpful to improve our understanding of μTV-environment.

Supplementary discussion 3. Sorting out stable and metastable states of a compression-expansion isotherm: A quasi Maxwell construction

The illustration given in Fig. 2 of the main text shows that Eq. 28 of the main text gives not only the Gibbs free energy for stables states but also that for metastable states. For a given pressure, the stable state has the lowest Gibbs free energy, i.e., that on the black curve in Fig. 2. Here, we show that it is also possible to sort out stable and metastable states from the compression-expansion isotherm with the help of a "Maxwell Construction" to as shown in Fig. 3 in the main text.

Supplementary note 3.1. Calculation of the pressure at the crossing points of Gibbs free energy

From Eq. 28 in the main text, we obtain the following result for the pressure at the crossing point of the large and middle branches of Gibbs free energy (i.e., the first and the second lines of Eq. 28),

$$p_{c1} = -\frac{\left(2-e^{\beta\varepsilon}\right)\left[\beta\varepsilon+\ln\left(2-e^{\beta\varepsilon}\right)\right]}{2\left(e^{\beta\varepsilon}-1\right)^2} \quad . \tag{3.1}$$

The pressure at the crossing point of the narrow-pore and middle-pore branches is given by,

$$p_{c2} = \frac{\left(2-e^{\beta\varepsilon}\right)\left[\beta\varepsilon-\ln\left(2-e^{\beta\varepsilon}\right)\right]}{2\left(e^{\beta\varepsilon}-1\right)} \quad . \tag{3.2}$$

It is to note that there is a logarithm singularity on the RHS of Eq. (3.1) and Eq. (3.2). So, they give physically meaningful results only for $e^{\beta\varepsilon} < 2$. Since $\beta\varepsilon + \ln\left(2 - e^{\beta\varepsilon}\right) < 0$ when $e^{\beta\varepsilon} < 2$, Eq. (3.1) gives always a positive result under this condition. With the help of Eq. (29) in the main text, we can show that the large and the middle branches of the Gibbs free energy crosses in the cases of an attractive fluid-wall interaction for $e^{\beta\varepsilon} < 2$ and in the case of a repulsive fluid-wall interaction for any temperature. The condition for a hysteresis loop occurring in the compression-expansion isotherm near p_{c1} is $p^*(2d^+) < p^*(2d^-)$. Eq. (29) in the main text gives $p^*(2d^-) = 1/\left(2e^{\beta\varepsilon}\right)$ and $p^*(2d^+) = \left(2 - e^{\beta\varepsilon}\right)/2$. Although these results satisfy always the inequality $p^*(2d^+) < p^*(2d^-)$, $p^*(2d^+)$ is positive only for $e^{\beta\varepsilon} < 2$. The condition for a hysteresis loop occurring near p_{c2} is $p^*(d^+) < p^*(d^-)$. Eq. (29) in the main text gives $p^*(d^-) = 1$ and $p^*(d^+) = \left(2 - e^{\beta\varepsilon}\right)e^{-\beta\varepsilon}$. For a repulsive fluid-wall interaction, i.e., $0 < e^{\beta\varepsilon} < 1$, $p^*(d^+) < p^*(d^-)$ holds for any temperature while for an attractive fluid-wall interaction $1 < e^{\beta\varepsilon} < 2$, the inequality is not satisfied for any temperature (the narrow-pore and the middle pore branches of the Gibbs free energy does not cross in this case). Since only the illustrations for a repulsive fluid-wall interaction are given in the main text, we restrict our

consideration to this case in the following discussions.

Supplementary note 3.2. A quasi Maxwell construction

Now, we show analytically that a Maxwell construction allows for resorting out stable and metastable states in a compression-expansion isotherm, i.e., placing the horizontal lines corresponding to p_{c1} and p_{c2} so that the corresponding turquoise and pink zones have the equal area as illustrated by Fig. 3 of the main text. Let L^*_{1t} denote the intercepting point of the horizontal line p_{c1} with the middle branch of the compression-expansion isotherm (see Fig. 3 in the main text), which gives the lower integration limit for calculating the area of the turquoise zone. Using p_{c1} given by Eq. (3.1) and Eq. 29 in the main text (the second line on its RHS), we obtain,

$$L^*_{1t} = \frac{1}{p_{c1}} - \frac{2(e^{\beta\varepsilon}-1)}{2-e^{\beta\varepsilon}} = -\frac{2(e^{\beta\varepsilon}-1)^2}{(2-e^{\beta\varepsilon})[\beta\varepsilon+\ln(2-e^{\beta\varepsilon})]} - \frac{2(e^{\beta\varepsilon}-1)}{2-e^{\beta\varepsilon}} = -\frac{2(e^{\beta\varepsilon}-1)}{2-e^{\beta\varepsilon}}\left[1+\frac{e^{\beta\varepsilon}-1}{\beta\varepsilon+\ln(2-e^{\beta\varepsilon})}\right]. \quad (3.3)$$

The intercepting point of the horizontal line p_{c1} with the large pore branch of the compression-expansion isotherm (the first line on the RHS of Eq. 29 in the main text) gives the following result for the upper integration limit for calculating the area of the pink zone,

$$L^*_{1p} = \frac{1}{p_{c1}} - 2(e^{\beta\varepsilon}-1) = -\frac{2(e^{\beta\varepsilon}-1)^2}{(2-e^{\beta\varepsilon})[\beta\varepsilon+\ln(2-e^{\beta\varepsilon})]} - 2(e^{\beta\varepsilon}-1)$$

$$= -\frac{2(e^{\beta\varepsilon}-1)}{2-e^{\beta\varepsilon}}\left[2-e^{\beta\varepsilon}+\frac{e^{\beta\varepsilon}-1}{\beta\varepsilon+\ln(2-e^{\beta\varepsilon})}\right] . \quad (3.4)$$

The area of the turquoise zone below p_{c1} is given by,

$$S_{1t} = \int_{L^*_{1t}}^{2} dl\left[p_{c1} - \frac{1}{l+2(e^{\beta\varepsilon}-1)/(2-e^{\beta\varepsilon})}\right] = p_{c1}(2-L^*_{1t}) - \ln\left[2+\frac{2(e^{\beta\varepsilon}-1)}{2-e^{\beta\varepsilon}}\right] + \ln\left[L^*_{1t}+\frac{2(e^{\beta\varepsilon}-1)}{2-e^{\beta\varepsilon}}\right]$$

$$= p_{c1}(2-L^*_{1t}) - \ln\left[\frac{2}{2-e^{\beta\varepsilon}}\right] + \ln\frac{2(e^{\beta\varepsilon}-1)^2}{(2-e^{\beta\varepsilon})[-\beta\varepsilon-\ln(2-e^{\beta\varepsilon})]} . \quad (3.5)$$

The area of the pink zone above p_{c1} is given by,

$$S_{1p} = \int_{2}^{L^*_{1p}} dl\left[\frac{1}{l+2(e^{\beta\varepsilon}-1)} - p_{c1}\right] = p_{c1}(2-L^*_{1p}) + \ln[L^*_{1p}+2(e^{\beta\varepsilon}-1)] - \ln[2+2(e^{\beta\varepsilon}-1)]$$

$$= p_{c1}(2-L^*_{1p}) - \beta\varepsilon - \ln 2 + \ln\frac{2(e^{\beta\varepsilon}-1)^2}{(2-e^{\beta\varepsilon})[-\beta\varepsilon-\ln(2-e^{\beta\varepsilon})]} . \quad (3.6)$$

$$S_{1t} - S_{1p} = p_{c1}(L^*_{1p} - L^*_{1t}) + \beta\varepsilon + \ln(2-e^{\beta\varepsilon}) . \quad (3.7)$$

From Eqs. (3.3), (3.4) and (3.1), we obtain,

$$L^*_{1p} - L^*_{1t} = \frac{2(e^{\beta\varepsilon}-1)}{2-e^{\beta\varepsilon}}[1-2+e^{\beta\varepsilon}] = \frac{2(e^{\beta\varepsilon}-1)^2}{2-e^{\beta\varepsilon}} , \quad (3.8)$$

$$p_{c1}(L^*_{1p} - L^*_{1t}) = -\frac{(2-e^{\beta\varepsilon})[\beta\varepsilon+\ln(2-e^{\beta\varepsilon})]}{2(e^{\beta\varepsilon}-1)^2}\frac{2(e^{\beta\varepsilon}-1)^2}{2-e^{\beta\varepsilon}} = -[\beta\varepsilon+\ln(2-e^{\beta\varepsilon})] . \quad (3.9)$$

Eqs. (3.7) and (3.9) lead to $S_{1t} = S_{1p}$.

Now, we show that the area of the turquoise zone below p_{c2} is also equal to the area of the pink zone above p_{c2} (see Fig. 3 in the main text). The lower and upper integration limits are given respectively by,

$$L_{2t}^{*} = \frac{1}{p_{c2}} = \frac{2(e^{\beta\varepsilon}-1)}{(2-e^{\beta\varepsilon})[\beta\varepsilon-\ln(2-e^{\beta\varepsilon})]} \quad , \tag{3.10}$$

$$L_{2p}^{*} = \frac{1}{p_{c2}} - \frac{2(e^{\beta\varepsilon}-1)}{2-e^{\beta\varepsilon}} = \frac{2(e^{\beta\varepsilon}-1)}{(2-e^{\beta\varepsilon})[\beta\varepsilon-\ln(2-e^{\beta\varepsilon})]} - \frac{2(e^{\beta\varepsilon}-1)}{2-e^{\beta\varepsilon}} = \frac{2(e^{\beta\varepsilon}-1)}{2-e^{\beta\varepsilon}}\left[\frac{1}{\beta\varepsilon-\ln(2-e^{\beta\varepsilon})} - 1\right] \ . \tag{3.11}$$

The area of the turquoise zone below p_{c2} is given by,

$$S_{2t} = \int_{L_{2t}^{*}}^{1} dl \left[p_{c2} - \frac{1}{l}\right] = p_{c2}(1 - L_{2t}^{*}) + \ln\frac{2(e^{\beta\varepsilon}-1)}{(2-e^{\beta\varepsilon})[\beta\varepsilon-\ln(2-e^{\beta\varepsilon})]} \ . \tag{3.12}$$

The area of the pink zone above p_{c2} is given by,

$$S_{2p} = \int_{1}^{L_{2p}^{*}} dl \left[\frac{1}{l+2(e^{\beta\varepsilon}-1)/(2-e^{\beta\varepsilon})} - p_{c2}\right] = p_{c2}\left(1 - L_{2p}^{*}\right) + \ln\left[L_{2p}^{*} + \frac{2(e^{\beta\varepsilon}-1)}{2-e^{\beta\varepsilon}}\right] - \ln\left[1 + \frac{2(e^{\beta\varepsilon}-1)}{2-e^{\beta\varepsilon}}\right]$$

$$= p_{c1}\left(1 - L_{2p}^{*}\right) + \ln\frac{2(e^{\beta\varepsilon}-1)}{(2-e^{\beta\varepsilon})[\beta\varepsilon-\ln(2-e^{\beta\varepsilon})]} - \ln\frac{e^{\beta\varepsilon}}{2-e^{\beta\varepsilon}}$$

$$= p_{c1}\left(1 - L_{2p}^{*}\right) + \ln\frac{2(e^{\beta\varepsilon}-1)}{e^{\beta\varepsilon}[\beta\varepsilon-\ln(2-e^{\beta\varepsilon})]} \ . \tag{3.13}$$

$$S_{2t} - S_{2p} = p_{c2}\left(L_{2p}^{*} - L_{2t}^{*}\right) + \beta\varepsilon - \ln\left(2 - e^{\beta\varepsilon}\right) \ . \tag{3.14}$$

From Eqs. (3.10), (3.11) and (3.2), we obtain,

$$p_{c2}\left(L_{2p}^{*} - L_{2t}^{*}\right) = -\frac{(2-e^{\beta\varepsilon})[\beta\varepsilon-\ln(2-e^{\beta\varepsilon})]}{2(e^{\beta\varepsilon}-1)}\frac{2(e^{\beta\varepsilon}-1)}{2-e^{\beta\varepsilon}} = -\left[\beta\varepsilon - \ln\left(2 - e^{\beta\varepsilon}\right)\right] \ . \tag{3.15}$$

Eqs. (3.14) and (3.15) lead to $S_{2t} = S_{2p}$. The above results show that it is possible to determine graphically the crossing points of the G-p plot from the compression-expansion isotherm, i.e., the p-V plot (V: volume). However, we prefer to call this procedure a quasi Maxwell construction since the two end points of the discontinuous volume jump do not have the same chemical potential. Thus, they are not two coexisting phases of a first-order phase transition.

Supplementary references

1. Dong, W. Thermodynamics of interfaces extended to nanoscales by introducing integral and differential surface tensions. *PNAS*, **118**, e2019873118 (2021).

2. Bender, C. M. & Orszag, S. A. *Advanced Mathematical Methods for Scientists and Engineers*, Springer Science+Business Media (1999).

3. Varnik, F., Baschnagel, J. & Binder, K. Molecular dynamics results on the pressure tensor of polymer films. *J. Chem. Phys.* 113, 4444 - 4453 (2000).
